# Legume genome structures and histories inferred from *Cercis canadensis* and *Chamaecrista fasciculata* genomes

**DOI:** 10.1111/tpj.70981

**Published:** 2026-06-10

**Authors:** Hyun‐oh Lee, Jacob S. Stai, Qiaoji Xu, Thulani Hewavithana, Pratheesh Soman, Rabnoor Batra, Alex Liu, Brandon D. Jordan, Rachel Walstead, Jerry Jenkins, Melissa Williams, Jenell Webber, Jane Grimwood, John T. Lovell, Tomáš Brůna, Shengqiang Shu, Keykhosrow Keymanesh, Joanne Eichenberger, Jeremy Schmutz, David M. Goodstein, Kerrie Barry, David Sankoff, Lingling Jin, James H. Leebens‐Mack, Steven B. Cannon

**Affiliations:** ^1^ ORISE Fellow, USDA‐ARS Corn Insects and Crop Genetics Research Unit 819 Wallace Rd Ames Iowa 50011 USA; ^2^ Department of Mathematics University of Ottawa Ottawa Ontario K1N 6N5 Canada; ^3^ Department of Computer Science University of Saskatchewan Saskatoon Saskatchewan S7N 5C9 Canada; ^4^ School of Computer Science University of Waterloo Waterloo Ontario N2L 3G1 Canada; ^5^ USDA—Agricultural Research Service Corn Insects and Crop Genetics Research Unit 819 Wallace Rd Ames Iowa USA; ^6^ Genome Sequencing Center, HudsonAlpha Institute for Biotechnology Huntsville Alabama 35806 USA; ^7^ US Department of Energy Joint Genome Institute Berkeley California 94720 USA; ^8^ Department of Biology University of Georgia Athens Georgia 30602 USA

**Keywords:** allopolyploidy, Caesalpinioideae, Cercidoideae, *Cercis canadensis* (redbud), *Chamaecrista fasciculata* (partridge pea), Fabaceae, legumes, whole genome duplication

## Abstract

The legume family originated ca. 60–65 million years ago and soon diversified into at least six lineages (now extant subfamilies). The signal of whole genome duplications (WGD) is apparent in species sampled from all six subfamilies. The early diversification has posed difficulties for resolving the legume backbone structure and the timing of WGDs, especially in Caesalpinioideae where the diversification and WGD signals coincide. In this study, we report the genome sequences and annotations for *Cercis canadensis* (Cercidoideae) and *Chamaecrista fasciculata* (Caesalpinioideae) to help resolve the timings of WGDs relative to subfamily origins and the ancestral legume karyotype. Analyses of genome assemblies from four subfamilies within Fabaceae show that the last common ancestor of all legumes likely had seven chromosomes, with a genome structure similar to the extant *Cercis* genome. The retained karyotype structure, the lack of a WGD in the last 100+ Mya (*Cercis* and the lineage leading to it following the eudicot γ whole‐genome triplication), and the unusually slow rates of nucleotide substitution and structural evolution in the *Cercis* genome underscore its utility as a genomic proxy for the last common ancestor of all legume species. Our analysis supports an allopolyploid origin of Caesalpinioideae, with progenitors from lineages along the backbone of the legume phylogeny. Rapid diversification and the inferred allopolyploid origin of Caesalpinioideae provide a partial explanation for the difficulty in resolving the backbone of the legume phylogeny and early Caesalpinioideae diversification.

## Significance Statement

Polyploidy has been a common theme in flowering plant evolution and in the 60–70‐million‐year history of the legume family. Comparative analyses including new, chromosomal genome assemblies for *Cercis canadensis* and *Chamaecrista fasciculata* indicate that whereas *C. canadensis* ancestors have not experienced polyploidy since the core eudicot gamma (γ) triplication approximately 135 Mya, independent whole genome duplications have occurred in the ancestry of Leguminosae subfamilies Papilionoideae, Caesalpinioideae, Dialioideae and tribe Bauhinieae within Cercidoideae.

## INTRODUCTION

The legume family (Leguminosae or Fabaceae) is the third largest family of flowering plants, comprising about 770 genera and 19 500 species (Lewis et al., 2013; LPWG, 2017). Its species play a crucial agronomic role as an important source of protein for a large proportion of the global human population, and in fixing nitrogen (N_2_) from the atmosphere and converting it to reduced forms that are used by plants to produce amino acids and other biomolecules (Chu et al., 2004; Herridge et al., 2008; Köpke & Nemecek, 2010; Martins et al., 2003; Salvagiotti et al., 2009). Legumes originated within the fabid (rosid I) angiosperm clade toward the end of the Mesozoic era, around 60–70 Million Years Ago (Mya) (Bruneau et al., 2008; Koenen et al., 2021; Lavin et al., 2005; Magallón et al., 2015; Zhao et al., 2021), potentially within 1 or 2 million years before or after the Cretaceous–Paleogene (K‐Pg) boundary; and within a few million years of this origin, the family had radiated into six subfamilies: Cercidoideae, Detarioideae, Dialioideae, Duparquetioideae, Caesalpinioideae, and Papilionoideae (LPWG, 2017; https://www.zotero.org/google‐docs/?hnJCSQ). Most agronomically important species (and approximately two thirds of species in the family) fall within the Papilionoid clade (Gepts et al., 2005; Cardoso et al., 2012; LPWG, 2017), but the other subfamilies contain many species of great importance in term of ecological service and economic value, including timber species (e.g., *Gleditsia*, *Caesalpinia*), forage (e.g., *Acacia*, *Leucaena*), and human consumption (e.g., *Tamarindus*, tamarind; *Detarium*, sweet detar; *Ceratonia*, carob; *Tylosema*, marama bean) (Cannon et al., 2011; Chen et al., 2024).

This rapid initial diversification burst has made resolution of the legume phylogeny difficult, with the most recent publication of the Legume Phylogeny Working Group leaving it partly polytomous (LPWG, 2017). Further complicating the understanding of the genomic evolution in the family is the presence of apparently independent whole genome duplications within each subfamily (Cannon et al., 2015; Stai et al., 2025; Zhao et al., 2021). Based on chromosome counts, Goldblatt (1981) hypothesized that the base chromosome number of all legumes was likely *n* = 7. Genome duplications subsequent to the initial legume diversification would be consistent with that hypothesis. Although chromosome numbers in the family vary widely, there are clear modal counts for most subfamilies of *n* = 12 for Detarioideae and *n* = 14 for Cercidoideae, Dialioideae and Caesalpinioideae (Goldblatt, 1981; Ren et al., 2019). A chromosomal count has not been reported for *Duparquetia orchidacea* Baill., but polyploidy has been inferred from gene phylogenetic analysis (Stai et al., 2025). In Papilionoideae, the range of chromosome numbers is broad, but the majority of species in this subfamily have counts of *n* = 7–11, and genera such as *Cladrastis*, *Myroxylon*, and *Swartzia*, representing lineages that diverged early from the core Papilionoideae clade, have counts of *n* = 13–14 (Goldblatt, 1981; Ren et al., 2019).

Two recent studies with good sampling coverage across legume subfamilies have found agreement on a common legume backbone phylogeny (Koenen et al., 2020, 2021; Zhao et al., 2021): ((((Pap., Cae.), Dia.), *Dup*.), (Cer., Det.)), but alternative models that vary slightly from this have nevertheless continued to appear in the literature (setting aside *Duparquetioideae*, which is not included in our study or in all studies below): a model with Cercidoideae sister to the remaining subfamilies (Aygoren Uluer et al., 2020; Stai et al., 2019; Zhong et al., 2022); a model with Dialioideae sister to Caesalpinioideae (Baker et al., 2022); a model with Duparquetioideae sister to Dialioideae (Zuntini et al., 2024); and most recently, a model with a Detarioideae + Cercidoideae clade sister to a clade with Duparquetioideae and Dialioideae as successively sister to a Caesalpinioideae + Papilionoideae clade (Zhang et al., 2026). Additionally, although studies at the broadest level agree that the evolutionary history of the legumes has been marked by whole genome duplication (WGD), disagreement continues regarding the precise number and timing of these duplications. Koenen et al. (2021) found, on the basis of a gene count methodology, independent autopolyploidy at least the base of each of Papilionoideae and Detarioideae, in agreement with previous research (Cannon et al., 2015), but suggested that the Papilionoideae WGD was most likely preceded by another allopolyploid WGD shared with Caesalpinioideae and Dialioideae, discarding previous hypotheses of a Caesalpinioideae‐specific WGD. This latter allopolyploid WGD hypothesis was derived from the Gene‐tree Reconciliation Algorithm with MUL‐trees for Polyploid Analysis (GRAMPA) (Thomas et al., 2017). Zhao et al. (2021) were in agreement with Koenen et al. (2021) regarding the hypothesis that multiple rounds of WGD affect Papilionoideae; however, on the basis of counts of ‘bursts’ of duplicated genes at various nodes, they placed the other WGD affecting Papilionoideae at the crown node of all legumes. Zhao et al. (2021), however, did not discard the possibility of a Caesalpinioideae‐specific WGD; indeed, they concluded that each legume subfamily (except Duparquetioideae) bore evidence of independent WGDs, subtending the crown node of each, in addition to a pan‐legume WGD, with most subfamilies in their model having undergone two successive rounds of WGD.

Several recent studies have concluded that since the time of the gamma triplication ~135 Mya (Jiao et al., 2012; Magallón et al., 2015), the genus *Cercis* has not experienced a WGD, and is thus fundamentally homoploid with the legume progenitor (Sinou et al., 2020; Stai et al., 2019; Zhong et al., 2022), to the extent that it may be a good living model for said progenitor (Stai et al., 2019). Here, we present genomic data supporting that not only is *Cercis* homoploid with the legume progenitor, based on the very strong syntenic preservation between *Cercis* and the non‐legume *Quillaja saponaria* Molina; but it also appears that the broad syntenic state of the legume progenitor has survived relatively intact in both of these long‐diverged lineages, as one copy in *Cercis*, and in duplicate in *Q. saponaria*, a near outgroup to the legumes. Likewise, Stai et al. (2025) and Koenen et al. (2021) argue that the complex reticulation patterns within legume subfamilies can be understood as consequences of allopolyploidy. The subgenome relationships in the Caesalpinioideae in particular appear to be consistent with allopolyploid hybridization from divergent parental lineages (Stai et al., 2025). Here, we present genomic data in accordance with that hypothesis. These conclusions are based on the high‐quality genome assemblies and annotations for *Cercis canadensis* L. and *Chamaecrista fasciculata* (Michx.) Greene, which we describe in this paper. These species represent two of the six generally recognized legume subfamilies (Cercidoideae and Caesalpinioideae, respectively) and thus are well placed for helping to infer early events in the evolution of the legumes.

For analyses of chromosome structural and gene family evolution, we make comparisons among six sequenced legume genomes that represent the four largest legume subfamilies and also against four non‐legume outgroup species. Those outgroup species, in order from youngest to oldest shared ancestry with the legumes (The Angiosperm Phylogeny Group, 2009), are *Q. saponaria* (Quillajaceae, in Fabales with the legumes) (Aygoren Uluer et al., 2020; Bruneau et al., 2008; Reed et al., 2023), *Prunus persica* (L.) Batsch (Rosaceae, Rosales, in the fabids with the legumes), *Arabidopsis thaliana* (L.) Heynh (Brassicaceae, Brassicales, in the malvids, sister clade of the fabids within the rosid clade), and *Vitis vinifera* L. (Vitaceae, Vitales, sister to the clade of all other rosids).

Of the newly sequenced genomes reported here, *C. canadensis*, also known as the eastern redbud, is a deciduous ornamental tree native to eastern North America. The *Cercis* genus is eponymous for the Cercidoideae subfamily. There are approximately 10 species within the genus—three native to North America, six native to China and south‐central Asia, and one native to the Mediterranean region (Davis et al., 2002; Fritsch & Cruz, 2012). We chose to sequence *C. canadensis* as the established the lack of polyploidy in this genus (at least since the core eudicot γ genome triplication) makes this species of interest as a potential genomic proxy for the legume progenitor (Li et al., 2023; Stai et al., 2019). We compare the assembly of *C. canadensis* to a genome assembly of *C. chinensis* Bunge (Li et al., 2023) and evaluate the *C. canadensis* assembly and annotations in the context of other legume species.

*Chamaecrista fasciculata*, commonly known as partridge pea, is in the Caesalpinioideae legume subfamily. Partridge pea (*Ch. fasciculata*) is an annual plant common in prairies of eastern and central North America (Fenster, 1991; Govaerts et al., 2021; Marazzi, 2023) (Note that from this point forward, we use ‘*C*.’ as the abbreviation for *Cercis* and ‘*Ch*.’ as the abbreviation for *Chamaecrista*.). We chose to sequence *Ch. fasciculata* both as an accessible, experimentally tractable representative of the Caesalpinioideae, and because this genus exhibits symbiotic nitrogen fixation (SNF), hosting nitrogen‐fixing Bradyrhizobium bacteria in specialized structures (nodules) on roots. *Ch. fasciculata* has been used as a model for research on the ecology, physiology, and evolution of symbiotic nitrogen fixation (Singer et al., 2009). Most genera in the Papilionoideae exhibit SNF as do species in several clades within the Caesalpinioideae, but none of the other legume subfamilies. The presence and absence of SNF within the rosid nitrogen‐fixing clade (NFC; a sub‐clade of the fabids) has presented a puzzle: did SNF evolve early in the NFC, with subsequent loss from many lineages (Sprent et al., 2017)? Or did SNF arise independently (or semi‐independently, following evolution of key potentiating events) in numerous lineages? The former model (single origin, multiple loss) was supported by seminal reports (Griesmann et al., 2018; van Velzen et al., 2019). The latter model (independent or semi‐independent origins in 16 clades, with losses in approximately 10) is supported by an evaluation and modeling of SNF presence and absence across more than 12 700 species in the NFC (Kates et al., 2024).

## RESULTS

### Genome assembly and annotation assessment

Characteristics of final chromosome‐level assemblies for *C. canadensis* and *Ch. fasciculata*, for both resolved haplotypes, are given in Table 1. To assess the completeness of the genome and annotations, we performed BUSCO analysis (Benchmarking Universal Single‐Copy Orthologs; v. 5.7.1) using both fabales and eudicot reference databases (Manni et al., 2021). Using the eudicots reference set, the completeness of *C. canadensis* was 98.6 and 99.5% for the genome and annotations, respectively (Table 1). For *Ch. fasciculata* (also using the eudicots reference set), the completeness was 98.8% and 99.4% for the genome and annotations, respectively. Using the fabales comparison set (fabales_odb10), the corresponding values were lower (96.2 and 96.6% for *Cercis* and 93.4 and 93.9% for *Chamaecrista*), likely reflecting the fact that the fabales database includes only representatives from the Papilionoid subfamily, which evidently insufficiently represents the earlier‐diverging *Cercis* and *Chamaecrista* taxa. Notably, the proportions of duplicated BUSCOs are 2.4% for both *Cercis* haplotypes, and 39.0 and 7.1% for *Chamaecrista* haplotype 1 and 2, respectively (Table 1), providing additional support for the lack of a genome duplication in the *Cercis* lineages since the core eudicot gamma event and presence of one in a *Chamaecrista* ancestor.

**Table 1:** Genome and gene statistics for *Cercis canadensis* and *Chamaecrista fasciculata*. Haplotypes 1 and 2 in each case indicate the haplotype assembly for the respective genome. The BUSCO values are from the ‘genome’ mode, using eudicot reference set as noted (eudicots_odb10 lineage dataset, created 2024‐01‐08; Manni et al., 2021)

Species	*C. canadensis*		*C. fasciculata*	
Haplotype	1	2	1	2
Pseudochromosome number	7	7	8	8
Total scaffold length (bp)	342 014 377	315 499 427	580 459 023	564 372 522
No. of scaffolds	43	7	24	17
No. of contigs	56	20	110	107
N50 of scaffolds (bp)	48 286 772	43 568 431	75 709 764	71 910 870
N50 of contigs (bp)	27 822 107	26 270 282	11 464 191	9 553 364
L50 of contigs	5	5	17	19
L90 of contigs	14	12	54	60
GC Ratio (%)	36.31	35.75	35.35	35.22
No. of gene	27 440	26 713	29 074	28 859
BUSCOs—eudicots				
Complete—eudicots	2293/98.6%	2296/98.7%	2314/99.4%	2294/98.7%
Complete and single‐copy—eudicots	2237/96.2%	2241/96.3%	1406/60.4%	2130/91.1%
Complete and duplicated—eudicots	56/2.4%	55/2.4%	908/39.0%	164/7.1%
Fragmented—eudicots	24/1.0%	21/0.9%	2/0.1%	19/0.8%
Missing—eudicots	9/0.4%	9/0.4%	10/0.5%	13/0.5%
Total BUSCO groups searched ‐ eudicots	2326	2326	2326	2326

### Comparisons of the *C. canadensis* and *Ch. fasciculata* genomes with resources from related species

The haplotype‐resolved *C. canadensis* genome assembly described here is the second near‐complete assembly published from this genus. Comparisons between the *C. canadensis* genome described here (either haplotype) and the assembly for *C. chinensis* (Li et al., 2023) show the two assemblies to be similar in size and structure, but with rearrangements (inversions) on chromosomes 3, 5, and 6 (Figure S2) and exhibiting a mean identity of 93.76% in regions of alignment. The assembly sizes for the two species are similar: 352.8 and 342.0 Mbp for the total assembly sizes in *C. chinensis* and *C. canadensis*; and 331.8 and 340.3 Mbp for the chromosomally anchored sequences in *C. chinensis* and *C. canadensis*.

The median Ks distance between *C. chinensis* and *C. canadensis* is Ks = 0.026, or 0.013 to the common ancestor—in comparison with Ks of 0.076 from either *Cercis* species to the legume crown node (Figure 8d). Assuming a median date for the legume crown node of 63.3 Ma [BEAST analysis and fossil calibration times from Koenen et al. (2021)], and assuming a constant rate for *Cercis* from present to that date, *C. canadensis* and *C. chinensis* may have diverged approximately 10.9 Mya (63.5 Mya*[Ks 0.013/Ks 0.076]).

The haplotype‐resolved *Ch. fasciculata* genome assembly described here is the second assembly of this species—the first being a contig‐level assembly (Griesmann et al., 2018), for isolate NF‐2018‐5 (derived from line MN87), GenBank accession GCA_003254925.1, with scaffold N50 of 56.6 kb and total assembly length of 429.1 Mb. The chromosome‐scale assembly described here, of isolate ISC494698, has scaffold N50 of 75.7 Mbp (more than a thousand‐fold greater contiguity than the previous assembly) and total assembly length of 580.4 Mb.

### Synteny relationships show independent WGDs early in four legume subfamilies, excepting *Cercis*

**Figure 1:**
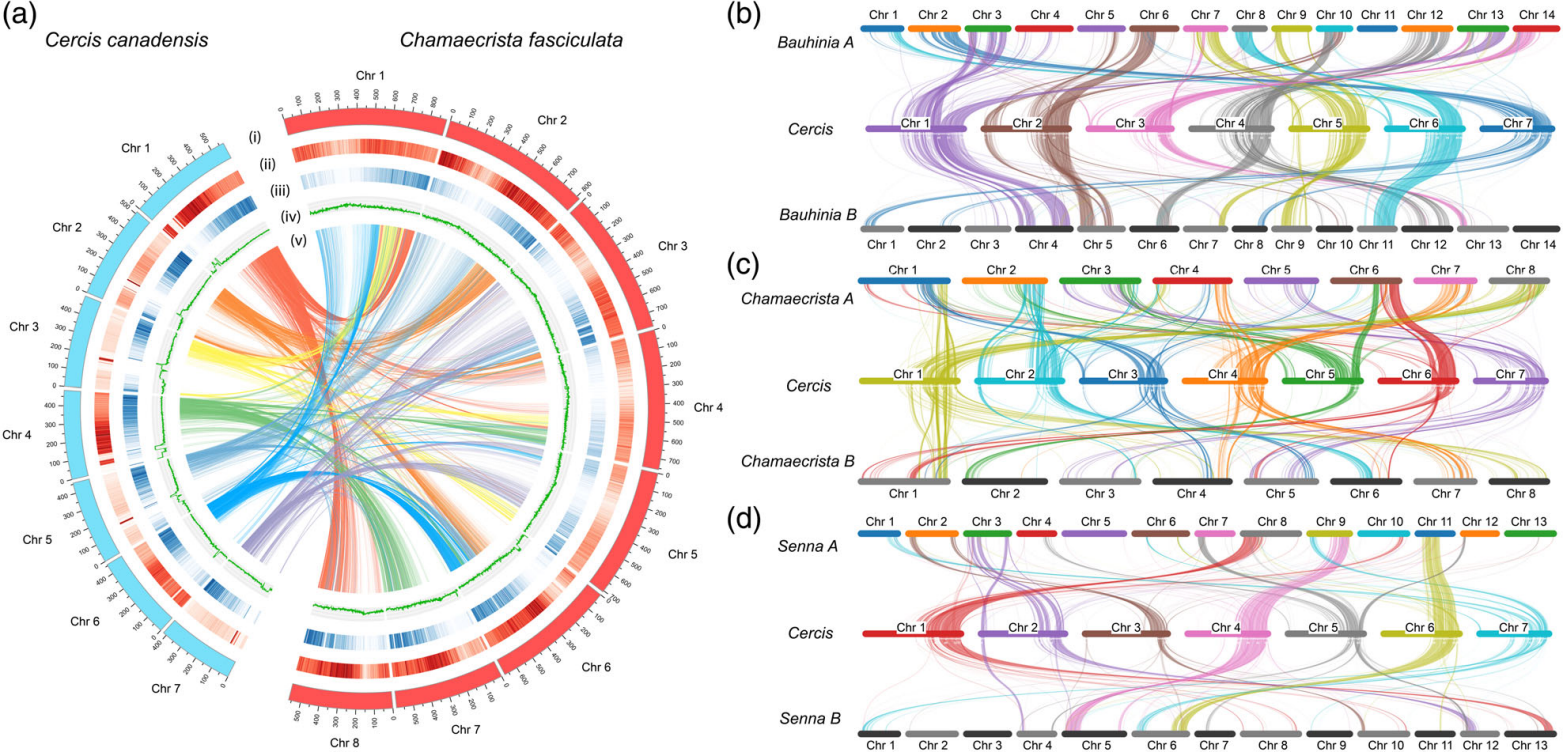
Plots of genomic features and syntenic relationships. (a) Circos plots representing features of the *C. canadensis* and *Ch. fasciculata* genomes. Circos track (i): chromosome length (Mbp); track (ii): repeat density; track (iii): gene density; track (iv): GC plot; inner connecting lines (v): synteny regions, identified with MCScanX. (b–d) Synteny plots shown in SyntenyLink (Bandi & Gutwin, 2020; Hewavithana et al., 2023) images, showing two subgenomes in *Bauhinia* (b), *Chamaecrista* (c), and *Senna* (d) relative to *Cercis* at the center of each panel and the least/most fractionated subgenome above/beneath *Cercis*.

**Figure 2:**
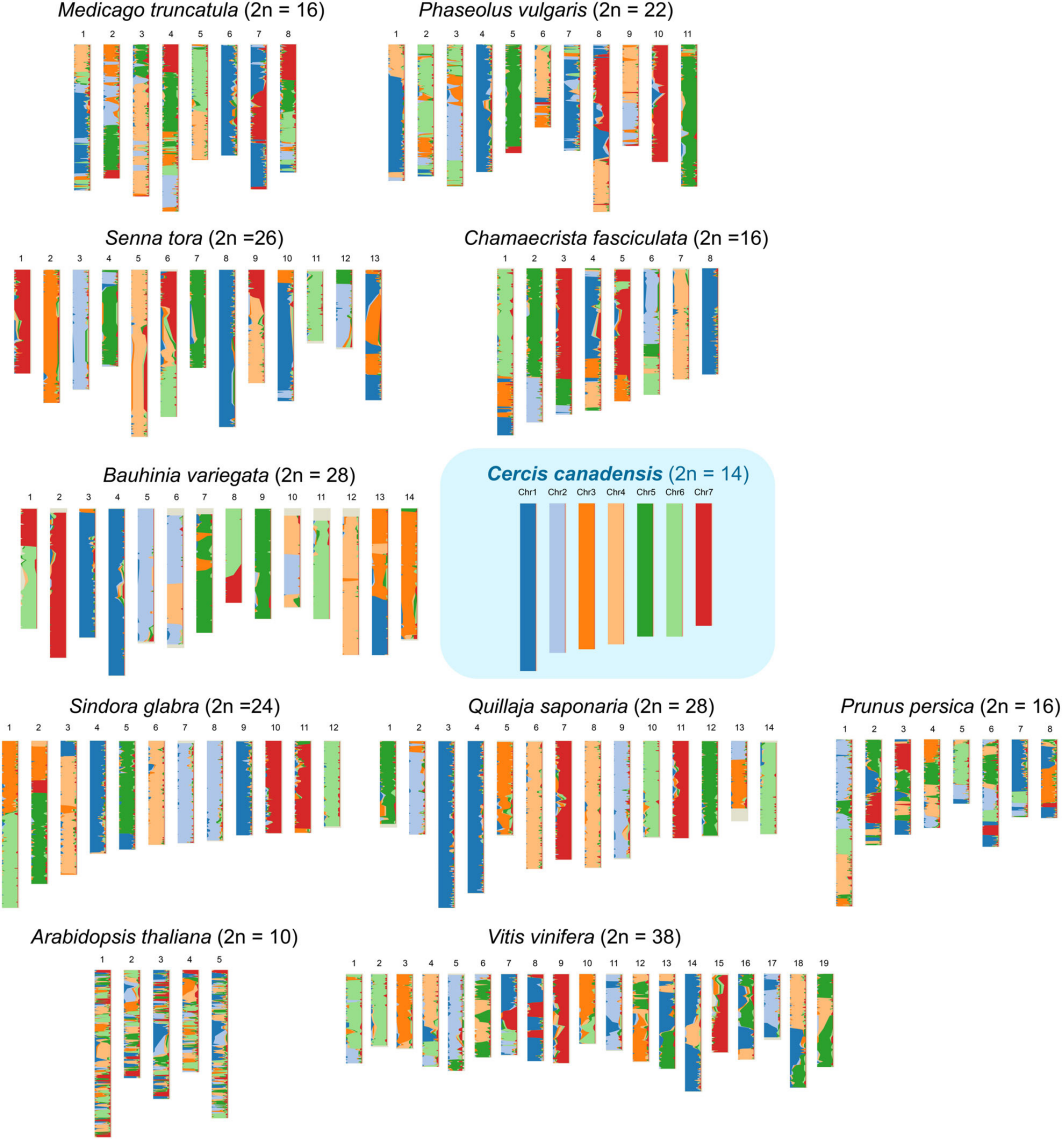
Synteny map of *Cercis canadensis* and comparison against legume and outgroup species. Chromosome‐level synteny maps were generated with the PanSyn program using *Cercis* as a reference relative to each indicated comparison species.

Synteny plots (Figures 1 and 2) show a general 1::2 pattern of chromosomal duplication between *Cercis* and other legume and close outgroup species. This pattern can be seen, for example, in *Cercis* chromosome 6 matching *Bauhinia* chromosomes 8A and 11B (Figure 1b), *Chamaecrista* chromosomes 6A and 1B, (Figures 1c), and *Senna* chromosomes 11A and 6B (Figure 1d).

Dot plot comparisons of *Cercis* and *Chamaecrista* to themselves and to each other (Figure S1), and synteny depths in the intra‐ and inter‐genome comparisons (Figure S1 legend), as well as *Ks* results (presented below), are consistent with a genome triplication in both *Chamaecrista* and *Cercis* in the timeframe of the ~135 Mya gamma event (Jiao et al., 2012), and a duplication near the origin of the Caesalpinioideae.

### Inference of a seven‐chromosome legume progenitor with genome structure similar to that of *Cercis*

Synteny and shared‐contig analyses suggest that the *Cercis* genome structure is similar to the hypothesized ancestral legume karyotype. The *Quillaja*, *Sindora*, *Bauhinia*, and *Senna* chromosome structures can all be represented in terms of doublings of the seven‐chromosome *Cercis* genome, with a small number of rearrangements in each lineage. When the chromosomes of different species are compared based on the chromosomes of *Cercis*, the combination of colors indicates chromosomal rearrangements (Figure 2). In *Quillaja* (a close outgroup to the legumes), the chromosomal correspondence with *Cercis* is a simple 1::2 match, with *Cercis* chromosomes generally matching two *Quillaja* chromosomes—often with chromosomal‐scale synteny across both species. The taxa requiring the fewest rearrangements relative to a simple doubling of *Cercis* (1*n* = 7) are *Quillaja* (1*n* = 14), requiring approximately 3 rearrangements; *Sindora* (1*n* = 12), requiring approximately 6 rearrangements; and *Senna* (1*n* = 13), requiring approximately 10 rearrangements. *Medicago* (1*n* = 8) and *Phaseolus* (1*n* = 11) show more complex restructuring—which would be consistent with the reduced chromosome counts from a hypothesized Papilionoid progenitor with 1*n* = 14 chromosomes (Ren et al., 2019).

**Figure 3:**
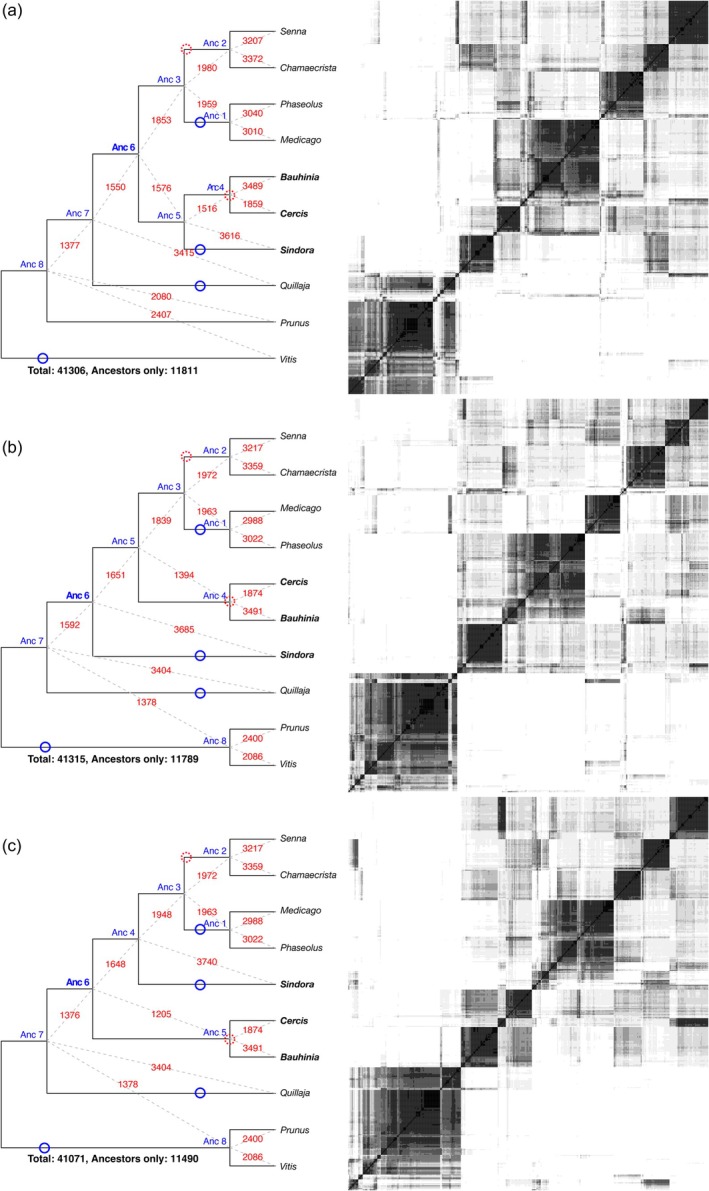
Phylogenetic relationships of 10 selected legume and dicot outgroup species, and heat maps of contig adjacencies representing inferred ancestral chromosome content. Left: three hypothesized phylogenetic relationships, representing evolutionary histories for which chromosome content is calculated. The legume ancestor node (Ancestor 6) is in bold. Dots indicate hypothesized or known locations of whole genome duplications: blue and red indicating probable auto‐ and allo‐polyploidies, respectively. Trees in a, b, and c represent the most frequently recovered phylogenetic models for these species—differing only in the relative placements of the Cercidoideae and Detarioideae subfamilies. The total Double Cut and Join (DCJ) distances between ancestral nodes suggest Tree 3 (c) as the most likely of these phylogenies by this method (lower values indicating lower distance or cost from cut and join operations). Right: Heat maps of the inferred legume ancestor (Ancestor 6 in the trees to the left), showing the clusters along the main diagonal of reconstructed contigs likely making up either six or seven ancestral chromosomes. Only the adjacencies at the ancestral node are shown here.

Heat maps (Figure 3) highlight syntenic contigs across the 10 indicated species (7 legumes and 3 outgroups), clustered by proximity under three hypothetical phylogenetic topologies. Given the hypothesized contig content of each chromosome, contigs are ordered along chromosomes using a Linear Ordering Problem routine. The clusters represent probable ancestral chromosomes, identified as syntenic contig groups co‐occurring in proximity at the indicated phylogenetic node within the included species and ancestors. We interpret these heat maps as indicating between 6 and 7 clusters, depending on density cutoffs, and we favor *n* = 7 for the legume progenitor. This conclusion is supported by (1) the 7::14 chromosomal relationships between *Cercis* and *Quillaja* and *Bauhinia* (Figures 1 and 2), and (2) the modal chromosome counts of 14 in Dialioideae, Caesalpinioideae, early diverging lineages of Papilionoideae, and (non‐*Cercis*) Cercidoideae (Figure 3; Ren et al., 2019). The modal count of *n* = 14 is readily explained by a single WGD from an *n* = 7 ancestor.

### Phylogenomic analyses and a consensus phylogenetic model of speciation and whole genome duplications

**Figure 4:**
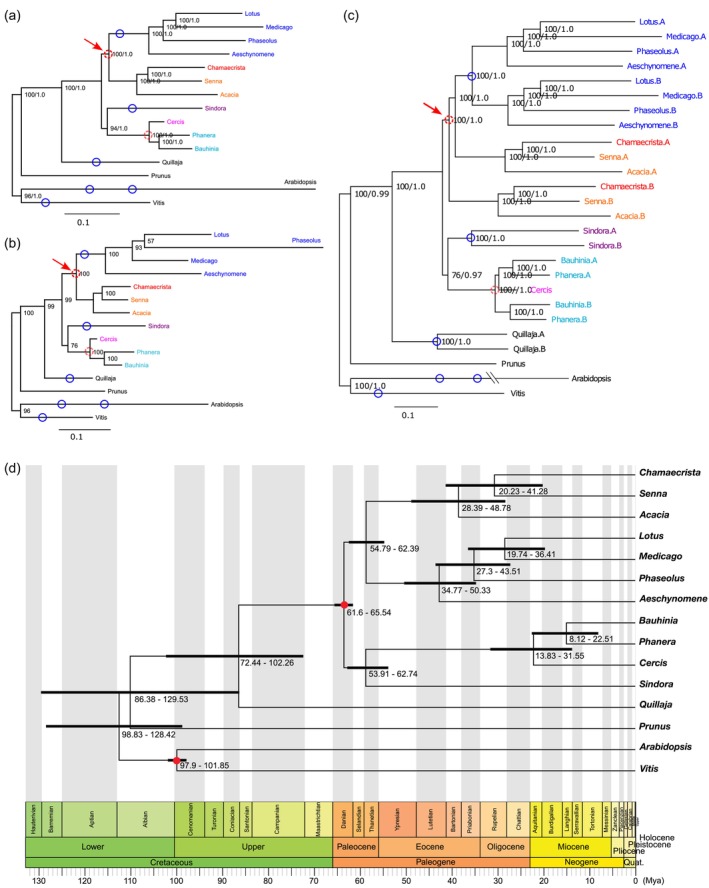
Species and subgenome phylogenies and time estimates for whole‐genome duplication gene families. (a) Consensus species tree with greatest likelihood (supermatrix analysis) and posterior probability (ASTRAL‐Pro3 analysis), based on 2212 low‐copy, species‐complete gene families. Divergences between WGD progenitors (predating allopolyploid events) are indicated with blue or red circles (from panel c; blue autopolyploidy; red allopolyploidy). Red arrow highlights the coalescent point of the Caesalpinioideae and Papilionoideae, for comparison with the gene family phylogeny in the next panel. Support values have the form 100/1.00; left side: bootstrap values reported by RAxML‐ng, based on resampling from a concatenated supermatrix alignment; right side: support values reported by ASTRAL‐Pro3. (b) tree based on the plastid *matK* gene; same symbols and coloring as in panel a. (c) Consensus gene family phylogeny, showing inferred whole genome duplications (WGDs), calculated from 250 gene families with near‐complete representation of species and WGD‐derived gene duplications, with species labeled as ‘A’ or ‘B’ per WGD clade to permit resolution of duplications in the phylogeny. Red circles indicate locations of inferred WGD events. Circles: inferred polyploidy events (colors indicating hypothesized type: blue auto‐, red allo‐, purple auto‐ or allo‐). Support values have the form 100/1.00; left side: bootstrap values reported by RAxML‐ng, based on resampling from a concatenated superalignment; right side: support values reported by ASTRAL‐Pro3, from 250 distinct trees calculated by RAxML‐ng. (d) The divergence time estimation of 15 taxa was generated using node calibration. The calculation was performed using BEAST (Bouckaert et al., 2014), and the blue bars at the nodes represent the 95% highest posterior density (HPD) interval. The red circles indicate the node calibration, which was set to 100 Mya for *Vitis*‐*Arabidopsis* and 63.5 Mya for the Legume crown node (Koenen et al., 2021). The geological time scale represents million years and was created using the R package strap (Bell & Lloyd, 2015).

The duplication and speciation histories for the legumes in this study are evident in gene families constructed from the proteomes from each species. From 250 gene families with few sequence losses or gains relative to known WGD events (see Materials and Methods section for filtering parameters), we recovered the same tree from ML analyses of a concatenated alignment of the 250 gene families, and a coalescence‐based analysis (ASTRAL‐Pro3; Mirarab & Warnow, 2015; Zhang & Mirarab, 2022), of the 250 gene trees (Figure 4a). The 250 selected families are representative of the full genomes in terms of chromosomal representations in each species, as evaluated by chi‐square tests for frequencies of chromosomes and species in the gene families and the full annotation sets (Data S7). An ML gene tree was also generated using a plastid *matK* gene alignment (Figure 4b). In addition, supermatrix and the coalescence‐based analyses of the subgenome labeled gene yielded the same topology (Figure 4c), revealing placements of speciation events and the progenitors of tetraploid *Quillaja*, Cercidoideae, Sindora, Caesalpinioideae, and Papilionoideae ancestors. The inferred timing of divergence between pairs of progenitor genomes is shown as circled nodes in the tree (Figure 4c).

Within the concatenated alignment (supermatrix) phylogeny, the basic species topology is generally congruent with the *matK*‐based phylogeny representing the plastome history (Figure 4a,b, respectively), and similar to results reported previously (Bruneau et al., 2008; Koenen et al., 2020, 2021; LPWG, 2017; Stai et al., 2025; Zhao et al., 2021). We note, however, a key topological difference between the species tree (Figure 4a,b) and phylogenomic phylogeny including subgenomes (Figure 4c). The species+subgenome phylogeny, analyzed including labeled homeologs, shows non‐monophyly in the two Caesalpinioideae progenitor clades and the two Cercidoideae WGD clades. The apparent non‐monophyly seen in the Caesalpinioideae Figure 4c is due to distinct gene histories of WGD‐derived homeologs. In our consensus phylogenies (Figure 4a–c), Cercidoideae and Detarioideae form a clade sister to a Caesalpinioideae + Papilionoideae clade as inferred by Zhang et al. (2026). This topology implies the fewest structural rearrangements as indicated by the lowest DCJ distance (Figure 3c). In Bruneau et al. (2008), Detarioideae and Cercidoideae form a grade, sequentially sister to the Caesalpinioideae‐Papilionoideae clade. In Koenen et al. (2021) and Stai et al. (2025), Detarioideae and Cercidoideae are sister clades. In Zhao et al. (2021), Detarioideae and Cercidoideae are reported as either sister to one another or unresolved (polytomous at the base of the family), or with Detarioideae sister to a clade including the remainder of the family. In LPWG (2017), these two subfamilies are reported as polytomous at the base of the family.

In the consensus species‐ and subgenome‐level phylogeny (Figure 4c), the placement of progenitors of the WGDs is apparent early in each of the four legume subfamilies included in this study, and several features in the Cercidoideae and Caesalpinioideae require discussion and interpretation. In the Cercidoideae, *Bauhinia* and *Phanera* are clearly ancient tetraploids, but not *Cercis*. However, in the consensus phylogeny (as well as in many individual gene phylogenies examined), *Cercis* groups with one of the *Bauhinia/Phanera* post‐duplication lineages rather than outside (and prior to) the inferred WGD. Consistent with reports from Stai et al. (2019), Li et al. (2023), and Stai et al. (2025), a plausible explanation for this topology is that the WGD in the Cercidoideae was allopolyploid in nature, with a progenitor of *Cercis* contributing one subgenome and a species in an ancestral sister clade to extant Cercidoideae contributing the other subgenome. Such a scenario could account for the high degree of species tree—gene tree discordance and the high reticulation index along the spine of the Cercidoideae clade in the ASTRAL estimate of the Leguminosae phylogeny reported by Zhang et al. (2026) based on analysis of 1559 low‐copy nuclear genes.

In the Caesalpinioideae, a WGD is clearly evident, resulting in homoeologous gene pairs in each species examined in this subfamily; yet the WGD progenitor lineages are sequentially sister to the Papilionoid clade. As with the Cercidoideae, allopolyploidy is a plausible explanation for this pattern. Specifically, the observed consensus species‐subgenome‐level topology (Figure 4c) is consistent with a speciation event followed by a significant period of divergence (several million years), and a subsequent allopolyploid merger of progenitor genomes that gave rise to Caesalpinioideae. One of the diploid lineages would then have gone on to become the progenitor of the Papilionoideae—which experienced its own WGD prior to diversification within that subfamily. Again, this scenario is consistent with high gene tree discordance and reticulation indices described by Zhang et al. (2026) for the spines of the Caesalpinioideae and Papilionoideae clades within their analyses of low‐copy nuclear genes to estimate the Leguminosae phylogeny. Going forward, there is potential for finer resolution of Papilionoid/Caesalpinioid WGD events with chromosomal reconstruction analyses including high‐quality assemblies of *Swartzia* and *Cladrastis* clades genomes (both outside of the core Papilionoideae). Ancestral chromosome reconstructions to date have focused on genomes within the core Papilionoideae (9 genera each in Wang et al. (2017) and Ren et al. (2019)). Based on available chromosome count data for 318 legume genera, Ren et al. (2019) report probable haplotype counts in the five larger subfamilies of 13–14 in Papiloidoideae, 14 in Caesalpinioideae, 14 in Dialioideae, 7 and 14 in Cercidoideae, and 12 in Detarioideae.

**Figure 5:**
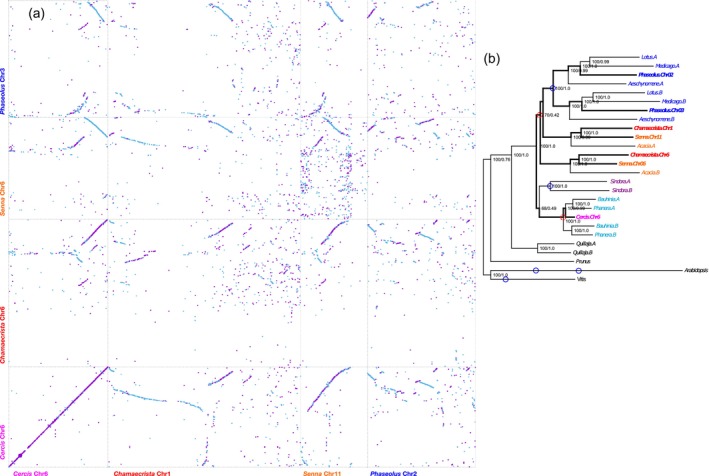
Genomic dot plots from chromosomes homoeologous and orthologous with *Cercis* chromosome 6, and consensus phylogenetic tree from gene families drawn from those chromosomes. (a) The plot shows comparisons among paralogous chromosomes in each of *Chamaecrista*, *Phaseolus*, and *Senna*, and also comparisons with *Cercis*. Chromosomes on the X axis are *Cercis* 6, *Chamaecrista* 1, *Senna* 11, and *Phaseolus* 2. Chromosomes on the Y axis are *Cercis* 6, *Chamaecrista* 6, *Senna* 6, and *Phaseolus* 3. (b) Consensus phylogeny derived from gene families with homologs present on all chromosomes in (a). Gene families were selected that had orthologs and paralogs from each of these chromosomes, and that also had (1) all 15 species present, (2) two paralogous genes in at least 8 of 11 legume species, and (3) had consistent outgroup rooting. For the four indicated species, paralog labeling was derived from the chromosomes of the respective genes. For the other species, ‘A’ and ‘B’ labels were applied based on examination of the trees. Species in bold correspond by color with those in the dot plot.

**Figure 6:**
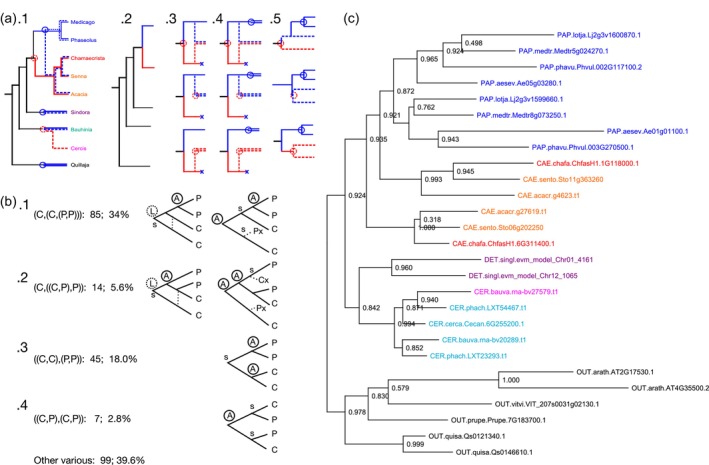
Schematic of gene, species, duplication paths, showing inferred allo‐ and autopolyploid events, and a gene family example. (a) A model of gene family and species evolution under allopolyploidy, illustrating the way that a collection of distinct gene family topologies can result from allopolyploidy followed by stochastic gene conversion or other subgenome interactions. From left to right: a.1 shows several possible paths (distinguished by color and line patterns) that may be observed for genes deriving from allopolyploidy. a.2 shows the initial speciation event, giving rise to a blue and a red gene (each from a distinct species). a.3–a.5: Circles show the locations of apparent WGDs (note that WGDs can appear at different depths, depending on subgenome interactions following allopolyploidy). a.3 shows the several gene family configurations following allopolyploidy and further elapsed time; dotted lines indicate distinct paralogs (red and blue dotted lines, top), or gene conversion (two dotted blue lines, middle or dotted red lines, bottom). a.4 shows the gene configurations after further elapsed time. a.5 has the gene families rearranged to give a typical resolved depiction, as would be produced by phylogenetic software. Any of the three topologies could be observed among different gene families following allopolyploidy. (b) Counts of phylogenetic patterns in the Caesalpinioideae (CAE) and Papilionoideae (PAP). The cartoons in b.1–b.4 show configuration of clades of CAE (C) and PAP (P) sequences, from 250 scored gene families, showing patterns of whole genome duplications. Letters ‘A’ and ‘L’ stand for inferred allopolyploid and autopolyploidy; and ‘s’ for speciation. Trees on the left are consistent with allopolyploidy affecting CAE, and those on the right are consistent with autopolyploidy affecting CAE—though requiring additional lineage losses, as indicated with ‘Cx’ and ‘Px’. (c) Example of an observed phylogeny, showing typical configurations of duplications within subfamilies. This is Legume.fam3.00878, a serine–threonine protein kinase family. This family is also one with sequences from the chromosomes and subgenomes shown in Figure 5.

The hypothesized allopolyploid origins of the Caesalpinioideae and the Cercidoideae can be seen in the consensus phylogeny in Figure 4(c), in the sytalog‐based phylogeny (Figure 5b) and, schematically, in Figure 6(a). In the schematic, an early speciation in the lineage leading to the Caesalpinioideae and Papilionoideae is represented as a red circle (Figure 6a.1). After significant divergence (Figure 6a.2,a.3), merger of two descendant diploid species (1*n* = 7) would have given rise to the 1n = 14 Caesalpinioideae. Following allopolyploidy, two subgenomes would be present in all ancestrally tetraploid species. In Figure 6(a.5), those distinct subgenome histories are represented as dotted or solid paths leading back to the last common ancestor of the two subgenomes (red circle).

### Tests of alternate phylogenetic models

We tested the occurrence of key features in the set of gene trees used for calculation of the consensus gene family phylogeny. Counts of four clade configurations for Caesalpinioideae and Papilionoideae are shown in Figure 6(b). The configurations (C,(C,(P,P))) and (C,((C,P),P)) (Figure 6b.1,b.2) are both consistent with allopolyploidy impacting Caesalpinioideae, followed by autoploidy at the base of the Papilionoideae. Together, these patterns account for 34% + 5.6% = 39.6% of the scored families. The second most common pattern is ((C,C),(P,P)) (Figure 6b.3), at 18%. This is consistent with independent WGDs in the CAE and PAP lineages. The least common pattern, with 2.8% of the trees, is ((C,C),(P,P)) (Figure 6b.4). This last pattern is consistent with a single shared WGD predating the CAE‐PAP split. It should be noted the most frequent patterns, (C,(C,(P,P))) and (C,((C,P),P)), could also be explained by sequential autoploidy events, but only if there had also been losses of particular affected lineages in many trees, as shown with ‘Px’ and ‘Cx’ in Figure 6(b). The allopolyploidy scenario is both more parsimonious and better supported by other analyses.

We also counted instances of gene clade ‘siblingness’ and ‘non‐siblingness’ in the Papilionoideae and Caesalpinioideae, testing whether the ratios of these topologies were similar in the two subfamilies. The exemplar gene phylogeny in Figure 6(c) illustrates ‘non‐sibling‐ness’ for Caesalpinioideae, and ‘sibling‐ness’ for Papilionoideae. The genome‐wide counts (Data S9) were recorded in a two‐by‐two table and for a simple chi‐square test of whether the observed rate of gene clade siblingness (Auto; A) to gene clade non‐siblingness (Allo; L) in the Papilionoideae are similar to the corresponding patterns in the Caesalpinioideae. The null hypothesis is that the proportions of A:L ratios are similar in the two subfamilies. Taking the Papilionoideae as providing the expected A:L ratio, is the observed A:L ratio in Caesalpinioideae significantly different? The *P*‐value for this test was 5.05E‐78, indicating there is a near‐zero chance of a lineage with an evolutionary history like that for Caesalpinioideae creating the rate of gene clade non‐monoploidy observed in Papilionoideae.

We also counted phylogenetic patterns in the Cercidoideae. Counts of two clade configurations were noted for the three species included in this subfamily (Data S9), using the same 250 gene families as for the counts above. A topology of (((Bau,Pha),Cer),(Bau,Pha)) was noted as ‘L’, signifying an allopolyploid pattern, with *Cercis* being sister to one of two homoeologous clades of *Bauhinia* and *Phanera*, indicating speciation followed by a later merger in the lineage sister to *Cercis*. A topology of (Cer,((Bau,Pha),(Bau,Pha))) was noted as ‘A’, signifying an autopolyploid pattern, with a WGD affecting the lineage sister to *Cercis* but not involving *Cercis*. The counts of these patterns were 73.6% L (Allo), 20.0% A (Auto), and 6.4% other.

### Divergence time estimation

The divergence time estimation is helpful in interpreting evolutionary events in the context of significant geological periods, and in evaluating the time scales over which events in the allopolyploid origins of Caesalpinioideae may have occurred (specifically, the divergence of progenitor genomes and subsequent polyploid event). We used node calibration points from Koenen et al. (2021) (Pentapetalae 100 and Leguminosae 63.5 mya; Figure 4c) and Magallón et al. (2015) (Fabaceae 93.2, Quillajaceae 96.3, and Pentapetalae 123.7 mya). For the present study, the most noteworthy date estimations for this study are those for the subfamily divergences—all of which had nearly overlapping ranges in our BEAST (Bouckaert et al., 2014) analysis: for the legume crown: 61.6–65.5 Mya; for the Cercidoideae‐Detarioideae clade, 53.9–62.7 Mya; for the Papilionoideae‐Caesalpinioideae clade, 54.8–62.4 Mya. The result of subfamily divergence occurring within a limited time span is similar to those reported in previous studies (Bruneau et al., 2008; Koenen et al., 2021; Lavin et al., 2005). In terms of older nodes, the highest posterior density (HPD) for the species of Pentapetalae utilized as an outgroup exhibited a relatively broad range, suggesting that the actual divergence may have occurred earlier than the conditions imposed in this study.

### Ks and phylogenomic analyses indicate independent WGD events in at least four legume subfamilies, dramatic mutation rate differences in legume lineages, and lack of a WGD in *Cercis*

**Figure 7:**
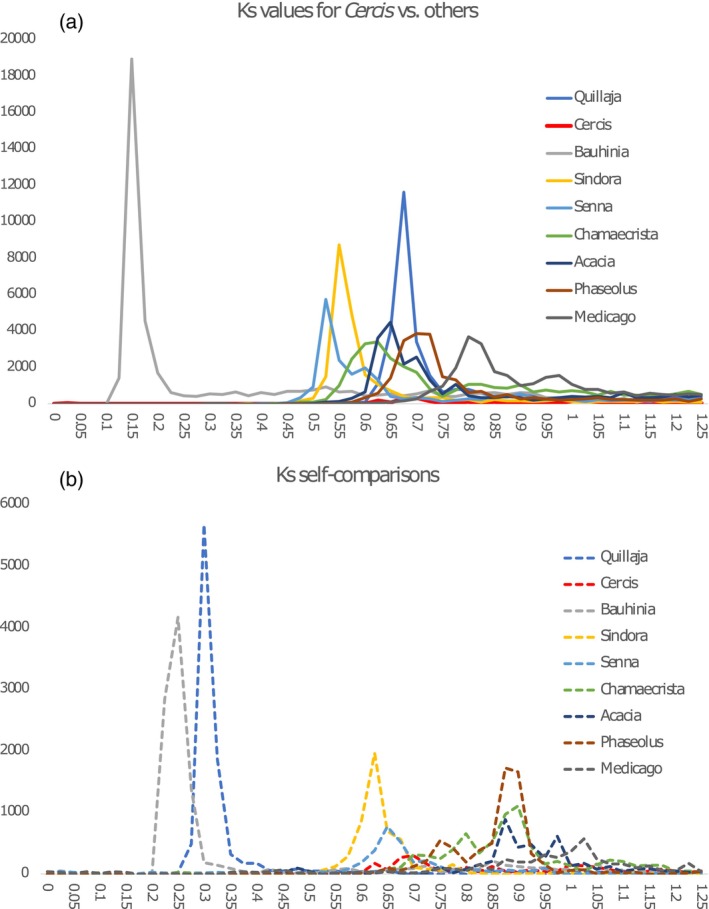
Ks distributions for selected species compared with *Cercis* and in self‐comparisons. (a) Comparisons between Cercis and other species (peaks representing speciations). (b) Ks values for species self‐comparisons (peaks representing WGD events).

**Figure 8:**
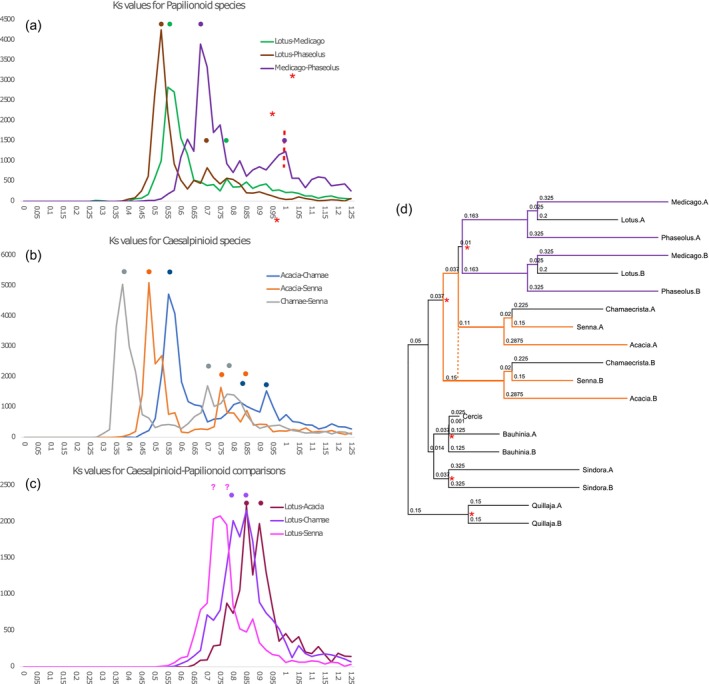
Ks distributions for Papilionoid and Caesalpinioid species‐pair comparisons, and phylogeny with Ks‐derived branch lengths. (a) Ks distributions for comparisons among species in the Papilionoideae: *Lotus japonicus, Medicago truncatula, Phaseolus vulgaris*. Dots show locations of Ks peaks—two for each species pair: the first corresponding to the speciation and the second corresponding with the papilionoid WGD. (b) Ks distributions for comparisons among species in the Caesalpinioideae: *Acacia crassicarpa*, *Chamaecrista fasciculata*, *Senna tora*. Dots show locations of Ks peaks—three for each species pair. The more recent peaks (Ks values 0.35–0.55) correspond with speciations. The older peaks (two for each species comparison, Ks values 0.7–0.9) represent the WGD at the base of the Caesalpinioideae, and possibly the inter‐subgenome exchange following allopolyploid merger (see main text for full description). (c) Ks distributions for a sample of Papilionoid and Caesalpinioid species. Peaks indicate the speciation Papilionoid–Caesalpinioid divergence, with doubled peaks possibly corresponding with allopolyploid origin of the Caesalpinioideae. (d) Phylogenetic topology species + subgenome tree in Figure 4(c), with branch lengths determined algebraically from Ks peaks, as a solution to a linear equation in which each internal and terminal branch is solved for, given the set of pairwise Ks values between all species and species self‐comparisons. The dotted line schematically represents one of two paths between homoeologous genes in Caesalpinioideae, consistent with the more recent of the two old Ks peaks in plot 8b. The older path represents speciation and the more recent one represents possible subgenome exchanges following allopolyploid merger.

Analyses of silent‐site mutations between species pairs can be used to examine relative evolutionary rates associated with both speciation events and WGDs. Although Ks plots are conducted pairwise between species, the dominant peaks from a set of species pairs can be interpreted in a phylogenetic context by solving for branch lengths from the system of equations whose values are stored in a matrix of distances between operational taxonomic units (OTUs). This is the same approach by which a neighbor joining tree can be constructed from a distance matrix. The plots in Figures 7 and 8 show Ks distributions for selected species pairs and also resolved algebraically relative to the consensus species topology (Figure 8d). The phylogeny with Ks‐based branch lengths has features consistent with allopolyploidy within Caesalpinioideae. The resolution in these plots is higher than typical because the Ks values are taken from the median Ks value per synteny block in the indicated species comparison, rather than from individual gene pairs; the use of median values decreases the impact of outlier Ks values from individual gene pairs.

In Figure 7(a), showing Ks values between *Cercis* genes and homologs in the indicated species (including *Cercis* compared with itself), the amplitude of the *Cercis‐*self plot is near zero in the Ks range shown. The peak for the *Cercis‐Cercis* comparison is near Ks = 1.8–2.0 (Data S10), consistent with the only polyploidy event in *Cercis* being much older than the origin of the legumes (presumably the core eudicot gamma genome triplication). The Ks peak with the smallest value is with *Bauhinia*, which is consistent with *Cercis* and *Bauhinia* being relatively close sister taxa within the Cercidoideae. All other peaks in Figure 7(a) are in the range 0.5–0.85, reflecting the substantial divergence with the other selected species, all of which are in other subfamilies (and another plant family all together in the case of *Quillaja*).

In Figure 7(b), showing Ks peaks for self‐comparisons for each species, *Cercis* is notable for exhibiting a Ks peak with substantially lower amplitude (several‐fold lower in amplitude than for the other species self‐comparisons). The Ks peaks for *Bauhinia* and *Quillaja* are both in small Ks bins (0.25 and 0.325, respectively), reflecting the similar recency of WGDs in those two taxa (WGDs that must be independent, since they are in different families).

In Figure 8(a–c), Ks peaks are shown for selected species comparisons: in Papilionoideae in 8a, Caesalpinioideae in 8b, and Papilionoideae‐Caesalpinioideae in 8c. In 8b, the plot for each species pair has an earlier dominant peak corresponding to the speciation node and a later, smaller peak corresponding with the earlier papilionoid WGD event. These peaks are highlighted with a dot above the respective peaks. For example, in the *Medicago‐Phaseolus* comparison, the speciation peak is at 0.7 and the WGD peak is at 1.0. Figure 8(b) is similar, showing comparisons among the three Caesalpinioid species included in this study; except in these comparisons, there is a strong primary peak reflecting speciations (*Acacia‐Chamaecrista*, *Acacia‐Senna*, and *Chamaecrista‐Senna*); and an intriguing possible doubled (bimodal) peak in each comparison at more distant (older) Ks bins: at 0.7 and 0.8 for *Chamaecrista‐Senna*, 0.75 and 0.85 for *Acacia‐Senna*, and 0.825 and 0.925 for *Acacia‐Chamaecrista*. For each species pair, these possible secondary double peaks are separated by 0.1 Ks units (‘possible’ here indicating the inherent uncertainty and limits of resolution in Ks plots). A secondary peak is expected to represent a WGD near the base of the Caesalpinioideae; but a doubled secondary peak may represent alternate evolutionary paths associated with allopolyploidy, as depicted in the schematic in Figure 8(d). Specifically, following allopolyploidy (Figure 6a.3), gene conversion or subgenome exchange may result in replacement of paralogs from one or the other constituent subgenomes (Bertioli et al., 2019; Gaeta & Pires, 2010). Over time, subsequent changes may occur, such as additional polyploidy in a derived lineage (Figure 6a.4). It is also possible that progenitor genomes could have acquired mutations at different rates prior to the allopolyploid merger. The resulting set of gene trees may have different topologies and timings (Figure 6a.5). The doubled Ks peaks around 0.70–0.95 seen in Figures 8(b,c) are consistent with this model—the older peak corresponding with the speciation prior to the allopolyploid merger and the more recent peak corresponding with subgenome interactions following the merger.

Because rates of silent‐site mutations may differ in different lineages, the values need to be considered in a phylogenetic context. Projecting the Ks values from the species pairs (Table S3) onto the consensus topology from Figure 4(c) and resolving the branch lengths algebraically gives the approximate branch lengths in Ks units. Phylogenetic analysis strongly supports a WGD at the base of the Papilionoideae and a separate allopolyploid event giving rise to the Caesalpinioideae—with one subgenome possibly derived from a papilionoid progenitor. Although Ks analysis alone is insufficient to make such inferences, the observed Ks peaks are consistent with this model of distinct WGDs in each of the examined subfamilies. Some lineages have apparently been evolving much faster than others (by the metric of silent‐site mutations), with *Medicago* having accumulated changes at nearly twice the pace of *Cercis* since their divergence from the common legume progenitor (Ks distances to the common ancestor of ~0.5 for *Medicago* and 0.225 for *Cercis*).

### Gene family expansion and contraction by comparing to representative species

**Figure 9:**
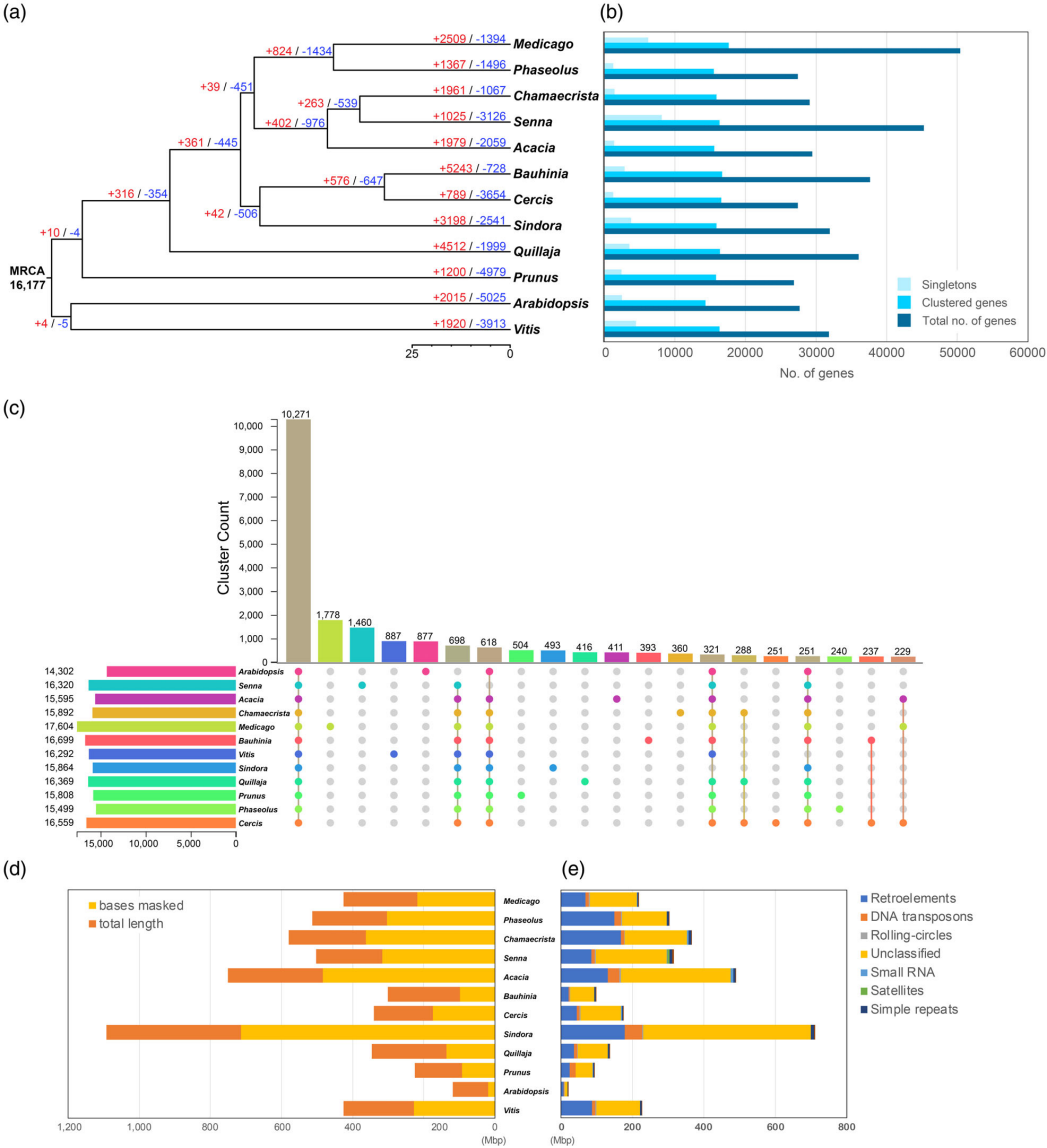
Genomic comparison of *C. canadensis* and *Ch. fasciculata* with related species. (a) Phylogenetic tree illustrating gene family size expansions and contractions across species. Red numbers indicate gene family expansions, while blue numbers denote contractions, both relative to the inferred MRCA gene family count, as estimated by CAFE5. (b) Bar graph comparing the total number of genes, clustered genes (orthogroup), and singleton numbers for the species used in the phylogenetic analysis. (c) Upset plot of clustered genes. The number clustered in all species is 10 271. (d) Comparison of repeat‐masked regions (orange) against total genome size (yellow) for each species. X‐axis in Mb. (e) Total base coverage of each repeat category across species (Table S2). Colors indicate distinct repeat types, as annotated by RepeatMasker. X‐axis in Mb.

Gene family expansions and contractions were analyzed using CAFE5, which employs a stochastic birth‐death model to estimate gene family size changes across a phylogeny. The numbers of expanded (red) and contracted (blue) gene families at each branch in (Figure 9a) reflect changes relative to the most recent common ancestor (MRCA) at each node. Gene family expansions indicate an increase in gene family size, while contractions represent a loss or reduction of gene families over evolutionary time.

At the subfamily level, Papilionoideae (represented by *Medicago* and *Phaseolus*) showed the largest number of gene family changes, with 824 expansions and 1434 contractions. At the species level, Bauhinia (Cercidoideae) exhibited the highest number of gene family changes (5243 expansions and 728 contractions), while *Cercis* showed the lowest (789 expansions and 3654 contractions), which aligns with the genome doubling in *Bauhinia* and with lower evolutionary rates in *Cercis* compared to other legumes, including *Bauhinia* within the same subfamily (Figure 9a).

Although the focus of this work is on the early diversification of legume genomes, as viewed through extant genomes and gene families, we briefly note some potential interpretations of the observed changes in Gene Ontology (GO) term enrichment above. GO enrichment analysis of expanded and contracted gene sets was performed to identify functional differences among species. Orthologous cluster analysis revealed that 10 271 clusters were common to all species, with 251 clusters unique to *Cercis* and 360 unique to *Chamaecrista* (Figure 9c). GO term annotation of *Cercis* genes (Figure S3) demonstrated a comprehensive representation of functional categories, including signal transduction, transcriptional regulation, and defense response. In the comparative GO enrichment analysis between *Cercis* and *Bauhinia* (Figure S5), *Cercis* demonstrated higher enrichment for pollen recognition and signaling‐related processes, while *Bauhinia* exhibited enrichment in stress‐response and transcriptional regulation pathways. Figure S4 provides a general summary of GO annotations for the entire gene set of *Chamaecrista*. In a comparison of *Chamaecrista* and *Senna*, both members of the Cassia clade, GO enrichment analysis of orthologous gene clusters unique to *Chamaecrista* (Figure S6a) identified significant enrichment in functions such as signal transduction, pollen recognition, auxin‐activated signaling pathways, and flavonoid biosynthetic processes. These functions were absent or underrepresented in *Senna* (Figure S6b), supporting a pattern of functional differentiation consistent with the presence of symbiotic nitrogen fixation in *Chamaecrista* and its absence in *Senna*. A relationship between pollen recognition and pollen‐tube growth, and symbiont recognition and infection‐thread growth, have long been speculated (Doyle, 1998) and observed (Oldroyd et al., 2011; Yokota & Hayashi, 2011). Auxin‐activated signaling is important in SNF during infection thread growth and nodule organogenesis (Oldroyd et al., 2011). Briefly: flavonoids released by the plant trigger production of Nod factor signaling molecules by the rhizobial partners. Upon recognition of the bacterial lipo‐chitooligosaccharide signaling molecule, a complex signaling and developmental process is initiated in the plant. Among other processes, auxin transport inhibitors are activated during the pre‐nodule infection stage, triggering nodule primordial cell division at the nodule site (Brown et al., 2001; Liu & Murray, 2016). Enhancement in gene sets involved in signal transduction in *Chamaecrista* may be related to additional host‐symbiont signaling involved in SNF—for example, in the flavonoid‐mediated signaling that is essential for determining host range specificity and promoting nodule development (Hassan & Mathesius, 2012; Subramanian et al., 2007). Thus, the enrichment of the auxin‐activated signaling pathway in *Chamaecrista* is consistent with the well‐established role of flavonoids in inhibiting auxin transport to facilitate nodule organogenesis (Weston & Mathesius, 2013) (Figure S6). However, it must be acknowledged that GO term enrichment offers only a coarse‐grained functional perspective. The SNF‐related patterns identified in this study are therefore considered preliminary and will be explored in greater detail—including gene‐level expression and functional analyses—in a separate study currently in preparation.

### Centromeric arrays in *Cercis* and *Chamaecrista* suggest divergent evolutionary histories

To further investigate structural evolution and chromosomal conservation across legume lineages, we examined centromeric repeat arrays in the two species. The arrays of probable centromeric repeats (consensus centromeric repeat sequences: Supporting Methods and Results: Data S1) are strikingly large in *C. canadensis*, extending up to 10.5 Mb (4.8 Mb average), and comprising approximately 9.86% of the total genome size. In contrast, the centromeric arrays in *Ch. fasciculata* comprise approximately 4.2% of the total genome size (repeat density plots in Figures S7 and S8 and genome self‐comparisons among the haplotype assemblies in Figures S9 and S10). As expected given their divergence time (~65.5 MYA to the common ancestor), the centromeric arrays in *Cercis* and *Chamaecrista* generally occur in different (non‐syntenic) locations—the arrays in *Cercis* disrupting synteny with *Chamaecrista* and vice versa (Figure S11). The pattern of synteny disruption suggests that centromeric arrays have originated (or moved) subsequent to the divergence of the respective lineages—unsurprising, given the lability of centromeres (Scelfo & Fachinetti, 2023). The centromeric arrays in *C. chinensis* are also large, totaling 6.23% of the genome size (Li et al., 2023), but array sizes differ significantly per chromosome relative to *C. canadensis*, and the sequence of the repeat arrays has diverged. The predominant centromeric repeats in *C. canadensis* and *C. chinensis* are both 104 bases in length, with an average sequence identity of 85.6%.

## DISCUSSION

The primary contributions of this work are (1) reports of the high‐quality assemblies and annotations of the *C. canadensis* and *Ch. fasciculata* genomes, representative species in two of the six legume subfamilies; (2) confirmation of earlier inference that *Cercis* lacks genome duplications that are present in most other legume genera; (3) evidence that the last common ancestor of legumes did not experience any lineage‐specific whole genome duplication beyond the ancestral eudicot γ triplication; (4) inference of a likely chromosome number of *n* = 7 for the legume progenitor; (5) establishment that the *Cercis* genome is little‐rearranged relative to the inferred, seven‐chromosome legume progenitor; (6) strong evidence of allopolyploid ancestry for Cercidoideae (excluding *Cercis*) and Caesalpinioideae—the latter likely involving a diploid progenitor from the lineage leading to the Papilionoideae, and some other as‐yet not identified diploid progenitor.

An important general implication of allopolyploidy is that species relationships may not be represented as a bifurcating phylogeny. Rather, a reticulate network of species relationships may be necessary to understand a species phylogeny. Furthermore, particular genes may have followed different evolutionary histories due to effects such as incomplete lineage sorting (ILS) and exchanges between subgenomes, adding further complexity to phylogenetic reconstruction when considered alongside gene duplication, loss, and homeolog divergence commonly associated with allopolyploidy. The reticulate nature of the legume phylogeny—particularly with respect to the Caesalpinioideae—has likely contributed to the difficulty in resolving portions of the legume phylogeny. This was a prominent conclusion of Koenen et al. (2021), who characterized the legume origin as a ‘complex paleopolyploid phylogenomic tangle’. Our conclusions and interpretations regarding the placements and nature of the allopolyploid relationships differ somewhat, however, from those of Koenen et al. (2021) and Zhao et al. (2021). Both studies posit a WGD early in the history of the legume family, independent of later WGDs inferred for the early history of legume subfamilies. The clear finding of lack of polyploidy in *Cercis*, and the later independent WGD in at least the base of the Papilionoideae (and lack of evidence of two WGDs affecting all papilionoid species), essentially rules out the possibility of a WGD subtending and predating the family as a whole. Koenen et al. (2021) do offer basal allopolyploidy as a possible explanation of observed duplication patterns: ‘Results suggest either a pan‐legume WGD event on the stem lineage of the family, or an allopolyploid event involving (some of) the earliest lineages within the crown group, with additional nested WGDs subtending subfamilies Papilionoideae and Detarioideae’. Our results are consistent with the allopolyploid model—but placing that allopolyploid event after the initial diversification, affecting the Caesalpinioideae but not the Detarioideae or Papilionoideae. (The Dialioideae was not evaluated as part of this study, but was in Stai et al. (2025), who concluded that Dialioideae also bears indications of allopolyploidy, potentially involving both this subfamily and the Caesalpinioideae). There is also evidence of allopolyploidy in the Cercidoideae, but private to that subfamily, with *Cercis* being a candidate for one parent of the remaining allopolyploid members of the subfamily.

Methodologies based on counting bursts of duplicated genes at nodes of a bifurcating phylogenetic tree (Zhao et al., 2021) are fundamentally vulnerable to misassessing the number and type of whole genome duplications, unless the biology underlying hybridization is kept in mind during interpretation. This is because, at the moment of hybridization, the two subgenomes start out related to one another at the speciation node of the parental lineage. Any burst‐counting methodology, looking back retrospectively, will see in the hybrid lineage a burst of gene pairs created at the speciation node of the ancestor's parents; these are the ‘true homeologs’, or gene pairs descending one from each parental lineage (Doyle & Egan, 2010). However, as the nascent allopolyploid diploidizes, it may undergo homologous recombination, up to and including the replacement of chromosomal regions or even whole homoeologous chromosomes. Homoeologous exchange is reviewed in Deb et al. (2023) and Mason and Wendel (2020) and has been observed for example in *Tragopogon* (Chester et al., 2012), Rice (Han et al., 2025; Zhang et al., 2019), *Arachis* (Bertioli et al., 2019), and Brassica (Bird et al., 2021; Gaeta et al., 2007). Thus, any burst‐counting methodology, looking back retrospectively, may see a second (apparent) burst of gene duplications after the hybridization event; these are ‘false homeologs’, or gene pairs created post‐hybridization during diploidization, derived as duplicates of the lineage of a single hybrid parent. Thus, the two bursts of gene duplications observed by Zhao et al. (2021)—the proposed pan‐legume WGD and the lineage‐specific Caesalpinioid WGD—could potentially be accounted for in a single genomic event, provided that Caesalpinioideae is an allopolyploid lineage, descended from parental lineages which diverged prior to the legume crown node.

Time estimates for early diversification in the legumes are important in the present study for interpreting the nature of allopolyploidy in the Caesalpinioideae. If species diversification along the legume backbone occurred over a long time period (say, tens of millions of years), then an allopolyploid merger might be quite surprising. If, on the other hand, the diversification occurred over a shorter time frame, then a merger of the diploid progenitors of Caesalpinioideae might be less remarkable. Although our taxon sampling was too sparse to infer crown diversification times for any subfamily, the timings of the subfamily origins (Cercidoideae, Detarioideae, Caesalpinioideae, Papilionoideae) indicate origins with overlapping ranges—in particular, 54.8–62.4 Mya for the Papilionoideae‐Caesalpinioideae clade, compared with 61.6–65.5 for the legume crown. Thus, it is not so difficult to imagine speciation of diploid progenitors of what would, upon later allopolyploid merger, become the Caesalpinioideae—and one or more of the diploid progenitors then continuing as independent lineages. In the case of Caesalpinioideae, one of those lineages appears to have then diversified (after a WGD) to become the Papilionoideae. The other diploid progenitor might either have been lost or might be found in some other extant lineage. Examples of recent allopolyploids and their still‐extant diploid progenitors include tetraploid *Arachis hypogaea* L. (Bertioli et al., 2019), which has been shown to be a recent (~10 k year) merger of two (still‐extant) diploid progenitors *A. duranensis* and *A. ipaensis*, which are estimated to have diverged approximately 2 Mya (Bertioli et al., 2019). Similarly, allotetraploid cotton, *Gossypium hirsutum*, resulted from a merger 1–2 Mya of A and D genome cotton species, *G. arboreum* and *G. raimondii*, which diverged 5–10 Mya (Li et al., 2014; Wendel & Grover, 2015).

That *Cercis* lacks a whole genome duplication and may have a chromosome structure and karyotype similar to the legume progenitor is not new; indeed, as early as 1981, Goldblatt suggested an ancestral legume karyotype of 7 chromosomes (Doyle, 2012; Goldblatt, 1981; Ren et al., 2019). Additionally, Li et al. (2023) reported a genome assembly of another *Cercis* genome (*C. chinensis*). Our work extends these prior results, modeling genome structural arrangements from representatives of four legume subfamilies and from outgroup representatives to infer both a likely phylogenetic backbone and ancestral chromosome structures. Although we did not include Duparquetioideae nor Dialioideae in our analyses, resolution of a Detarioideae + Cercidoideae clade sister to a Caesalpinioideae + Papilionoideae clade is consistent with the Leguminosae phylogeny estimates recently published by Zhang et al. (2026).

Particularly useful in both the phylogenetic and genomic structural analyses is the availability of *Q. saponaria*, as one the closest outgroup families to the legumes (the other being Surinaceae, likely sister to Quillajaceae; Bruneau et al., 2008). The syntenic similarity between *Cercis* and *Quillaja*, documented in Figures 1 and 2, is remarkable. Whole chromosomal synteny can be clearly and readily identified in the 1::2 relationships between these genomes. The most parsimonious explanation of this strong syntenic similarity is strong syntenic preservation since the time of the most recent common ancestor of *Cercis* and *Quillaja* (Aygoren Uluer et al., 2020). If the most recent common ancestor of *Cercis* and *Quillaja* is indeed the Fabales MRCA, and if there has indeed been strong syntenic preservation in both lineages since the time of that species, then we can with high confidence reconstruct every ancestral species along the lineage from the Fabales MRCA to *Cercis*, including the legume MRCA, as having had very similar chromosomal states: 7 chromosomes, with very few between‐chromosome rearrangements relative to living members of the *Cercis* and *Quillaja* genera.

Adding this to previously adduced useful features of the genus *Cercis*, such as its slow rate of evolution and its homoploidy with the legume progenitor (Li et al., 2023; Stai et al., 2019; Zhong et al., 2022), it seems fair to say that *Cercis* is the best living model of the original legume genome currently known. The synteny, phylogenomic, and Ks analyses presented here reinforce the finding that *Cercis* bears no evidence of lineage‐specific whole genome duplication (WGD) subsequent to the γ triplication, thereby affirming its function as a genomic proxy for the ancestral legume karyotype. Although the constraint this finding imposes on the possible chromosomal states of the legume progenitor directly contradicts the conclusion of Zhao et al. (2021) that there exists a WGD shared by all legumes, our analysis remains in full accord with their specific findings of independent WGDs specific to each of the other four legume subfamilies that we examined in this study: the Papilionoideae, Caesalpinioideae, Detarioideae, and Cercidioideae.

Our time estimations are also consistent with previous reports (Koenen et al., 2021; Renne et al., 2013) that the legume origin may have coincided with the Cretaceous–Paleogene (K‐Pg) boundary, 61.6–65.5 Mya—and with the mass extinction event that marked the conclusion of the Cretaceous period. The post K‐Pg period may have been a period of increases in both speciation and WGD retention, as posited previously (Fawcett et al., 2009; Vanneste et al., 2014). After the initial divergence within legumes, the divergence between Papilionoideae and Caesalpinioideae is estimated at 54.8–62.4 Mya, which occurred during the Paleocene, a period characterized by global recovery and diversification following the K‐Pg event. A similar pattern was observed for the divergence between Detarioideae and Cercidoideae, which is estimated to have occurred between 53.9 and 62.7 Mya (Figure 4d).

The earliest divergence among major papilionoid clades likely took place during the Eocene, as suggested by (Lavin et al., 2005). However, much of the lineage‐level diversification within genera appears to have accelerated later—particularly through the Oligocene and Miocene—when dramatic climate transitions altered ecosystems worldwide (Cai et al., 2025). The shift from a greenhouse world to cooler, drier conditions likely created new ecological opportunities, shaping the expansion and adaptability of both the Papilionoideae and Caesalpinioideae. In contrast, evidence indicates that the Cercidoideae diversified relatively early in the Miocene, perhaps in response to warm and humid environments still present during that period (Figure 4d). These divergence time estimates underscore the close relationship between major geologic and climatic events in the Paleozoic and Mesozoic eras and the evolution of the legume lineage.

The genome assemblies and annotations presented here, together with other resources representing the four largest subfamilies in the legumes, permitted us to construct gene families that robustly capture the core genic complement of the family; these gene families in turn support the existence of allopolyploid genome duplication events in Caesalpinioideae, as well as in the non‐*Cercis* Cercidoids—as proposed in previous literature: (Stai et al., 2019; Zhong et al., 2022). Recent research has emphasized that auto‐ and allopolyploidy do not necessarily represent strictly discrete categories. For deep‐time polyploid events in particular, post‐duplication processes such as diploidization and homoeologous exchange can obscure signals of origin, rendering a continuum‐based interpretation more appropriate (Twyford et al., 2025). As predicted by the model of Caesalpinioid allopolyploidy, considerable discordance is seen in the placement of Caesalpinioid genes (Figure 6b). WGD‐derived paralogs from species such as *Chamaecrista* often do not resolve as sister to one another, but rather as alternately sister to genes from other subfamilies. Likewise, our Ks analysis (Figures 7 and 8) appears to show a two‐peak signature of polyploidy during the legume diversification era, which we interpret as reflecting the same two apparent bursts of gene duplication observed by Zhao et al. (2021); but consistent with a model of an allopolyploid merger followed by subgenome exchanges. Future studies when Dialioid and Duparquetioid genomes become available will be able to further test the WGD status of those lineages.

*Chamaecrista* is of particular interest due to its capacity for SNF—in contrast with many other lineages in Caesalpinioideae—including genera such as *Senna*, which is a sister genus within the Cassiinae tribe (and within the broader Mimosoideae–Caesalpinieae–Cassiinae (MCC) clade in Caesalpinioideae). Within Caesalpinioideae, nodulation is present in approximately nine lineages and absent in a comparable number (Kates et al., 2024; Sprent et al., 2017). For comparison, nodulation is present in most genera in the Papilionoideae and absent in the other four legume subfamilies. While Griesmann et al. (2018) argued for a singular evolutionary origin of nodulation with subsequent widespread losses across lineages, recent phylogenomic analyses by Kates et al. (2024) suggest that nodulation may have arisen independently as many as 16 times. We hypothesize that the pattern of scattered SNF in Caesalpinioideae could have been facilitated by the subfamily's proposed allopolyploid history. If one of the progenitor species lacked SNF (whether due to loss or non‐gain), and the other progenitor had SNF capacity, then the allopolyploid merger would have produced a new polyploid species with a greater diversity of genes related to that capacity. The subsequent diploidization process could then have played out in various ways, with descendant lineages being vulnerable to stochastic loss of genes crucial to SNF. Such a process could help explain the uneven distribution of SNF capacity in the Caesalpinioideae—per Kates et al. (2024), probably 15 clades with SNF and 14 without.

Although the work presented here did not focus primarily on nodulation, the availability of a high‐quality genome assembly and annotations for *Ch. fasciculata* are expected to be of use in such studies in the future. Characteristics that make *Chamaecrista* suitable as a model for examination of SNF (Singer et al., 2009) include that it is physically small, with a short generation time, can be outcrossed or selfed, and exhibits considerable diversity across the North American habitats in which it is found.

We have no compelling mechanistic explanation for the unusual stability and apparent slow evolution in *Cercis*—evidenced both in the chromosomal structural conservation with, for example, *Quillaja* and by the extraordinarily low accumulation of silent‐site mutations (e.g., Figures 4a–c and 8d). Although the underlying mechanisms remain uncertain, it is plausible that the relatively slow rates of nucleotide substitution observed in woody perennials, combined with infrequent chromosomal rearrangements, have contributed to the long‐term genomic stability evident (Smith & Donoghue, 2008). We speculate that the unusually large centromeric arrays (in *C. canadensis*, comprising approximately 9.9% of the total genome size), may be related to the stability of the chromosome structure over the ~65 million year history of legume evolution—as either cause or effect or both. In particular, centromeric arrays may tend to grow if undisturbed by rearrangements. We speculate that large centromeres may also aid in maintaining chromosome structure by providing ‘unmissable’ mitotic attachment points. The exceptionally large centromeric arrays could conceivably be explained by centromeric drive, as discussed in Talbert and Henikoff (2022). According to this model, satellite variants at the centromere can compete for their inheritance in asymmetric female meiosis by preferential inclusion in the egg rather than the polar body. Satellite expansion may recruit more cenH3 and kinetochore proteins that can provide a stronger connection at the centromere, favoring transmission to the next generation. On the other hand, the repetitive character of centromeric arrays is also capable of creating a complex DNA topology which, together with mechanical forces applied by the microtubule during meiosis, makes this a potentially fragile region of the genome (Scelfo & Fachinetti, 2023). Significant changes in centromere size and location have been observed, for example, among *Glycine max* accessions (Liu et al., 2023). The combination of fragility and the repetitive structure of centromeres have been proposed as explanations for rapid sequence diversification and for centromere expansion and contraction (Altemose et al., 2022; Miga & Alexandrov, 2021; Scelfo & Fachinetti, 2023). Nevertheless, the robustness of centromeric arrays may differ between organisms, depending on the functioning of the numerous genes involved in centromere maintenance. Such genes include DNA polymerases, helicases, topoisomerases, the SMC5/6 complex, and a set of proteins in the constitutive centromere‐associated network (CCAN) (Kurzbauer et al., 2018; Scelfo & Fachinetti, 2023). Among the genes in the CCAN, the FANCD2 protein (Fanconi anemia complementation group D2 protein), which helps repair DNA damage, has been reported to be present in many plants, including *Arabidopsis* and a large collection of grass genomes, but it appears to be absent in legume genomes (Yadav et al., 2020). Indeed, we find no genomic orthologs of this gene (AT4G14970 in *Arabidopsis*) in any of the 11 legume genomes in our study. (With tblastn of AT4G14970 against the genome sequences in our study, the best‐hit E‐values are all greater than 0.1). In contrast, there are orthologs in *Vitis, Prunus*, and *Quillaja*, all with E‐values below 1e‐50. It is conceivable that some difference in the processes that maintains the centromeres (by analogy to the absent FANCD2 gene—though not necessarily due to this gene in particular) leads them to be more stable (and larger) than in many plants; and in turn, that stability may have contributed to the relative structural stability of the *Cercis* genome. Alternatively, some other factor may be responsible for the genomic stability; and in the absence of frequent genomic rearrangements, it is possible that the centromeric arrays have been free to expand for some tens of millions of years.

## CONCLUSIONS

The work here describes the high‐quality genomes and annotations for *C. canadensis*, eponymous for the Cercidoideae legume subfamily; and for *Ch. fasciculata* in Caesalpinioideae. These species are well placed taxonomically to aid inferences about key features of legume evolution, including the legume ancestral karyotype and the respective timing of subfamily origins and WGDs early in the legume radiation. Both *Cercis* and *Chamaecrista* appear to have been involved in allopolyploidy, with *Chamaecrista* a descendant of a paleopolyploid hybridization event, and the progenitor of *Cercis* likely contributing one subgenome to an allopolyploid event that gave rise to the remaining species in the Cercidoideae. In the Caesalpinioideae, the preponderance of gene families and Ks analyses suggests merger of two species that diverged along the taxonomic grade leading to the Papilionoideae and then merged to give rise to the allopolyploid Caesalpinioideae. Such an allopolyploid merger early in the evolution of SNF may help to explain the very uneven pattern of SNF presence and absence across the diverse Caesalpinioideae. Finally, a finding of allopolyploidy during the origin of the legumes provides an important example of diversification that is not modeled sufficiently with a standard bifurcating phylogeny.

## MATERIALS AND METHODS

### *C. canadensis* and *Ch. fasciculata* tissue source, genome assembly and annotation

For genome sequencing of *Ch. fasciculata*, 34 g of young leaf tissue was collected from a single field‐grown plant that was subsequently deposited at the Iowa State University herbarium under accession ISC494698. This tissue was used for extraction of high molecular weight DNA. The ISC494698 accession was from a population maintained for 15 years in Ames, Iowa (42.034752, −93.627405). The Ames population in turn derives from another wild population in Minneapolis, MN (44.965358, −93.326428). For annotation, tissue for RNA extraction was collected from a single progeny of ISC494698, from the following tissues: open flower, flower bud, root, leaf.

For genome sequencing of *C. canadensis*, 40 g of young leaf tissue was collected from a mature tree on the Iowa State University campus (42.028452, −93.642875) in Ames, Iowa. A sample from this plant was subsequently deposited at the Iowa State University herbarium under accession ISC453364. This tissue was used for extraction of high molecular weight DNA. For annotation, tissue for RNA extraction was collected from ISC453364 and from progeny of this plant, from the following tissues: seedling root, flowers, leaves, flower buds, shoot.

Genome assembly and annotation methods for *C. canadensis* and *Ch. fasciculata* are described in supplementary Methods and Results (S1). Briefly, the genomes of both *C. canadensis* and *Ch. fasciculata* were initially assembled using PacBio CCS (Circular Consensus Sequence) reads, and then, the contigs were oriented and ordered using Hi‐C data. Heterozygous snp/indel phasing errors were corrected using PacBio and Illumina data. The 99.46–99.58% of the assembled sequences were assigned to chromosomes. Finally, *C. canadensis* was assembled into 7 pseudochromosomes and *Ch. fasciculata* into 8 pseudochromosomes. The chromosomes contained within each haplotype were assigned a numerical identifier in accordance with their physical length, with this numerical sequence determined based on phasing information derived from the assembly. Haplotype assignments were biologically arbitrary, but scaffolds comprising each haplotype are complementary such that each chromosomal sequence is near‐complete (Table 1).

### Analysis of synteny and duplicated subgenomes resulting from polyploid events

For synteny calculations as illustrated in Figure 1(a), we used MCScanX (Wang et al., 2024), with visualization by Circos (Krzywinski et al., 2009). The SyntenyLink algorithm (Hewavithana et al., 2023) was used to identify two subgenomes derived from ancient allotetraploidy events in the ancestry of *Chamaecrista, Senna*, and *Bauhinia*. SyntenyLink assesses differences in substitution and fractionation patterns in synteny blocks as homoeologous gene duplicates (syntelogs) diverge. The results are visualized using SynVisio (Bandi & Gutwin, 2020) showing syntenic blocks linking regions of the *Cercis* genome to homoeologous regions of the *Bauhinia* (Figure 1b), *Chamaecrista* (Figure 1c), and *Senna* (Figure 1d) genomes. We also used PanSyn (Yu et al., 2024) to ‘paint’ regions of a set of comparison genomes with the colors of a designated reference species—in this case, *Cercis* (Figure 2).

### Ancestral genome reconstructions and calculation of rearrangement distances

To reconstruct ancestral genomes and analyze genomic rearrangements along alternate hypothesized phylogenies (Figure 3), we used RACCROCHE (Chanderbali et al., 2022; Xu et al., 2020, 2023). RACCROCHE retains a ‘generalized’ set of candidate gene adjacencies (Yang & Sankoff, 2011), allowing for a specified number of spacer genes to account for gene loss in extant genomes. Unlike other approaches that employ a greedy (Bernard et al., 2025) or consensus (Sun et al., 2022) approach, RACCROCHE employs combinatorial optimization by constructing an adjacency graph and solving it for a maximum‐weight matching. This procedure automatically identifies the most strongly supported adjacencies in extant genomes, which are subsequently linearized as ancestral contigs. To minimize biases arising from large differences in contig lengths, we divided each contig into ‘*g*‐mers’ of at most *g* genes, generating a larger set of contigs with more comparable lengths. These contigs were then matched against the chromosomes of extant genomes, and the number of times any two contigs co‐occurred on the same chromosome and their relevant ordering were recorded. Finally, complete‐linkage clustering (Defays, 1977) and linear ordering problem (Grötschel et al., 1984) were applied on the contigs based on their co‐occurrence patterns which offers RACCROCHE its ability to reconstruct both ancestral structure and chromosome number. It is important to note that duplicated genes are mapped to the same adjacencies, so the reconstructed ancestral genome corresponds to the optimal ‘monoploid’ configuration. This hypothetically corresponds to a state which precedes whole genome duplication, segmental duplication and even most tandem duplications. *g* = 10, 15, 20, 25, 30 were empirically tested for gap statistics analysis to estimate the optimal number of ancestral chromosomes. Values of g were selected based on prior empirical evaluation (see Xu et al., 2023), which showed that this range provides a balanced resolution for adjacency detection while maintaining robustness to noise.

### DCJ distance inference of species relationships

The Double Cut and Join (DCJ) distance (Yancopoulos et al., 2005) is used to quantify the structural differences between two genomes. Smaller DCJ values indicate fewer rearrangements between the two genomes. We calculated the DCJ distances for all branches in the given phylogenetic tree, including those between ancestral genomes and between ancestral and extant genomes, using the UniMOG tool (Hilker et al., 2012).

### Phylogenomic analyses and taxon sampling choices

For phylogenomic analyses, we first calculated legume‐focused gene families using the CDS and protein sequences of 36 legume species in 21 genera and four legume subfamilies (Table S1), as well as four non‐legume outgroup genera. The resulting set of 39 981 gene families generated using these inputs, and 25 732 families containing at least 4 sequences and at least 2 distinct genera, should capture much of the legume genespace (though missing Dialioideae and Duparquetioideae, and still biased toward the Papilionoideae). The legume‐focused gene families were first constructed using the Pandagma gene family workflow (https://github.com/legumeinfo/pandagma; Cannon et al., 2024).

For close analysis of relative timings of speciations, genome duplications, and potential signals of allopolyploidy (Figures 4, 5, 6), we focused on 15 species—four from Papilionoideae, three from Caesalpinioideae, one from Detarioideae, three from Cercidoideae, and four outgroup species. For computationally intensive genomic structural analysis (Figures 1, 2, 3), we focused on 10 species (listed below)—two each from Papilionoideae, Caesalpinioideae, Cercidoideae; one from Detarioideae, and three outgroups.

The main objectives for the reduced taxon sets were as follows: (1) where possible, include multiple diverse representatives, with high‐quality genome assemblies and annotations, from each of the legume subfamilies (no genome assemblies were available for *Duparquetia* or Dialioideae at the time of writing), (2) include suitable non‐legume outgroup species, at a range of evolutionary distances to help identify distinct legume clades in gene families; and (3) use a sufficiently small taxon number to permit both visual assessment and computationally intensive analyses of topologies.

The 15 species used for the phylogenomic analyses, with brief rationales for inclusion, are as follows: (1) Papilionoideae: *Medicago truncatula* Gaertn. and *Lotus japonicus* Regel (from the Hologalegina/inverted repeat‐lacking clade), *Phaseolus vulgaris* L. (from the Millettioid/Phaseoloid clade; Doyle & Luckow, 2003; Stefanović et al., 2009), and *Aeschynomene evenia* C. Wright (from the Dalbergioid s.l. clade; Cardoso et al., 2020; of interest for its distinct nodulation type); (2) Caesalpinioideae: three relatively divergent species from the large Mimosoideae–Caesalpinieae–Cassieae (MCC) clade, sensu LPWG (2017): *Acacia crassicarpa* A. Cunn. ex Benth. (Mimosoideae) and *C. fasciculata* (Michx.) Greene and *Senna tora* (L.) Roxb., both from the Cassia clade—the former with symbiotic nitrogen fixation and the latter without (Bruneau et al., 2008; Sprent et al., 2017); (3) Detarioideae: *Sindora glabra* Merr. ex. de Wit. (this was the only available genome from this subfamily at the time of writing); (4) Cercidoideae: *C. canadensis* L., as a primary focus of this work; and as comparisons within the subfamily: *Bauhinia variegata* (L.) Benth. (Zhong et al., 2022) and *Phanera championii* Benth. (Lu et al., 2024); (5) Outgroups: we included *Q. saponaria* Molina (Reed et al., 2023) as one of the closest non‐legume relatives; then (at increasing evolutionary distances): *P. persica* (L.) Batsch, in Rosaceae; *A. thaliana* (L.) Heynh., in the Malvid clade; and *V. vinifera* L., representing the sister lineage to most other rosid lineages (Magallón et al., 2015).

The 15 annotation sets from the reduced taxon set were placed into the initial legume orthogroups by homology, using the pandagma ‘fsup’ workflow (Cannon et al., 2024), parameterized with identity ≥30% and coverage ≥40%. This produced a set of 25 005 gene families that were used for further phylogenomic analyses. The full orthogroup collection and the hidden Markov models for each family are available at https://data.legumeinfo.org/LEGUMES/Fabaceae/genefamilies/legume.fam3.VLMQ/.

Protein sequences from each family were aligned using famsa (Deorowicz et al., 2016). The alignments were modeled using hmmbuild from the hmmer package (Finn et al., 2011) to generate hidden Markov models (HMMs). The original gene family sequences were then realigned to the HMM for each family and then trimmed to the match‐states of the HMM. Phylogenetic trees were then calculated using FastTree v. 2.1 (Price et al., 2010). Selected gene trees (e.g., Figure 6c) were also calculated using RAxML, with 1000 bootstraps.

### Calculation of species and subgenome phylogenies

To calculate a species phylogeny (Figure 4a), we identified a set of low‐gene‐copy gene families and then used those to calculate a phylogeny from a concatenated supermatrix of the alignments and also calculated a consensus of individual trees accounting for the coalescent process using ASTRAL‐Pro3 v1.19.3.6 (Mirarab & Warnow, 2015; Zhang & Mirarab, 2022). Of the 25 005 gene families, 2212 were identified for calculating an ASTRAL species phylogeny, based on the requirements that (1) all 15 species in the study were present, and (2) at least 9 of 11 legume species had a single copy in the gene family. For the 2212 low‐copy families with multiple paralogs for a species, a single representative sequence was selected, retaining the sequence with the fewest gap characters in the gene family alignment. This process results in a set of gene family alignments with one sequence per species. For the supermatrix method, a concatenated alignment was generated from all 2212 gene families, with genes (including homeologs/paralogs) placed in consistent order. The alignment was sampled at modulo 13 (taking every thirteenth amino acid) to make phylogenetic calculations tractable. The resulting alignment matrix had 88 770 sites. The consensus phylogeny was then calculated using RAxML‐NG (Kozlov et al., 2019), using the raxml‐ng ‐‐all workflow, with model LG + FC + G8m{0.559216}. The low‐copy families, alignments, and trees are available in Data S4.

For the ASTRAL‐Pro3 v.1.19.3.6 species phylogeny estimated while accounting for the coalescent process, we first calculated phylogenies for the 2212 low‐copy families using FastTree v. 2.1 (Price et al., 2010). A consensus tree was then calculated from those trees (concatenated into one file) (Mirarab & Warnow, 2015; Zhang & Mirarab, 2022). The topologies produced by the supermatrix and coalescent methods were the same. Support values are indicated in Figure 4(a) at each node: RAxML bootstrap values on the left and ASTRAL‐Pro3 local posterior probabilities on the right, for example, 100/1.0.

### Estimation of species and subgenome phylogenies

To calculate a consensus phylogeny that captures both speciation and likely whole‐genome duplication events (Figure 4c), we used two methods: maximum likelihood analysis of concatenated supermatrix using (Kozlov et al., 2019), and a gene tree summary approach ASTRAL‐Pro3, v1.19.3.6 (Mirarab & Warnow, 2015; Zhang & Mirarab, 2022) with putative homeologs labeled A and B. We also made a statistical test of observed topologies based on counts of the occurrence of alternate topologies across individual gene trees, as described below in the section ‘Tests of alternate phylogenetic models’, using a chi‐square test on a 2 × 2 contingency table.

Both the supermatrix and the coalescent (i.e., ASTRAL) methods can accommodate labeling of paralogous sequences. In effect, labeling homeologs (e.g., A and B) permits both the supermatrix and the coalescent methods to treat paralogous sequences assigned to subgenomes as if they came from distinct species. We scored 250 gene families that were selected from ‘nearly‐complete’ families—that is, families containing the expected number of genes from each species under the assumption of retention following known WGDs and no additional WGDs. Specifically, the families were filtered such that (1) all 15 species in the study were present; (2) at least 8 of 11 legume species in the family had exactly two putative homeologs; and (3) the family had a single outgroup rooting. These criteria resulted in 705 families. From these, the first 250 families (by family ID) were scored and labeled. Given the numbering of the gene families by the Pandagma workflow (Cannon et al., 2024), larger families (families with more paralogs per species) generally have lower sequence IDs, so the first 250 families should tend to be larger than families with higher IDs. Paralogous genes within selected families were then labeled as follows.

Homoeologous genes from ancestrally tetraploid species in each of the 250 selected phylogenies were labeled A or B based on their WGD clade membership within gene trees. For example, a WGD early in Papilionoideae would have produced two clades that later diverged into clades each containing a *Medicago* paralog and a *Phaseolus* paralog, so the labeling would give Medicago.A and Phaseolus.A in one clade and Medicago.B and Phaseolus.B in the other clade. The A and B labeling was applied top to bottom in rooted trees that had been ordered relative to the outgroup, by the Order function in the Archaeopteryx tree viewer (Han & Zmasek, 2009). This places sister clades with more nodes above those with fewer nodes, which generally results in the better‐represented Papilionoid clade placed at the top, and Cercidoid clade near the bottom. In cases where one CAE clade was nearer to PAP, its sequences were assigned A and those from the more distal clade were assigned B, for example (B,(A,(A,B))). The A/B labeling should otherwise be neutral with respect to other aspects of clade arrangements. Labelling for each gene family are given in Data S5, table ‘genes in tree order’. For the supermatrix method, given these labeled gene families, a concatenated alignment was generated from all 250 gene families, with genes and paralogs placed in consistent order: Lotus.A first, Medicago.A second, etc. The alignment was sampled at modulo 5 (taking every fifth amino acid) to make phylogenetic calculations tractable. The resulting alignment matrix had 27 974 sites. The consensus phylogeny was then calculated using RAxML‐NG (Kozlov et al., 2019), using the raxml‐ng ‐‐all workflow, with model LG + FC + G8m{0.623058}. The wgd‐copy families, alignments, and trees are available in Data S6.

Accounting for the coalescent process, we used ASTRAL‐Pro3, v1.19.3.6 (Mirarab & Warnow, 2015; Zhang & Mirarab, 2022) with labeled input trees for each of the 250 gene families estimated using FastTree v. 2.1 (Price et al., 2010). The topologies produced by the supermatrix and coalescent methods were the same. Support values are indicated in Figure 4(a) at each node: RAxML bootstrap values on the left and local posterior probabilities on the right, for example, 100/1.0.

### Tests of phylogenies derived from syntenic blocks

To evaluate whether signals of allopolyploidy seen in paralogous genes in the Caesalpinioideae correspond with chromosomal histories (specifically, whether homeologs from subgenomes show similar phylogenetic topologies to one another), we analyzed gene families with genes on a set of chromosomes with a high degree of synteny in the *Cercis*, *Chamaecrista*, *Senna*, and *Phaseolus* genomes (Figures 1 and 5). As was done for the larger phylogenomic analyses, we filtered the gene families such that: (1) all 15 study species in the were represented; (2) at least 8 of 11 legume species in the family had exactly two paralogs (possible homeologs); and (3) the family had a single outgroup rooting. This yielded 36 gene families (Data S7 and S7). We used the chromosomes as the basis for labeling the genes from those four species: *Cercis* 6, *Chamaecrista* 1 and 6, *Senna* 11 and 6, and *Phaseolus* 2 and 3 (Figure 5). For species other than the four indicated ones, paralogous genes were labeled ‘A’ or ‘B’, as indicated above. From those 36 gene families, a concatenated supermatrix of 18 252 positions was derived and used for calculating a phylogeny with raxml‐ng ‐‐all workflow, with model LG + FC + G8m{0.561922}. A consensus tree was also calculated from the 36 trees (concatenated into one file) using ASTRAL‐Pro3, v1.19.3.6 (Mirarab & Warnow, 2015; Zhang & Mirarab, 2022). The topologies from the supermatrix and coalescent approaches were the same. Support values are indicated in Figure 5(b). The gene families, alignments, and trees are available in Data S8.

### Tests of alternate phylogenetic models

For statistical test of occurrences of phylogene allopolyploid versus autopolyploid patterns in the Papilionoideae (PAP) and Caesalpinioideae (CAE) clades, we scored the 250 selected trees, giving a 2 × 2 matrix (‘allo’ or ‘auto’ configuration, for CAE and PAP sequences), and then used a chi‐square test that calculates whether the ratio of PAP autopolyploid versus allopolyploid topologies is similar to the ratio of CAE auto‐ versus allo‐topologies. We also scored the 250 trees relative to the most common observed topologies for the PAP and CAE WGD clades, as well as the topologies observed in the CER subfamily. Scorings for each gene family are given in Data S9, worksheets ‘genes in tree order’ and ‘allo‐auto test’.

### Synonymous‐site (Ks) calculations

Ks values (Figures 7 and 8) were calculated as part of the pandagma gene family workflow (Cannon et al., 2024). For a given species pair, gene pairs were identified first based on homology using mmseqs2 (Steinegger & Söding, 2017), then filtered based on inclusion in synteny blocks identified by DAGChainer (Haas et al., 2004). Ks values for those gene pairs were then calculated using PAML (Yang, 2007). A median value was then calculated for all genes in a synteny block. For the purpose of calculating Ks frequencies, median values for the genes in the block were applied to all gene pairs in that block, which were in turn used to calculate genome‐wide Ks value frequency distributions (Figures 7 and 8). Median Ks values for each species pair (both speciation and WGD peaks) were used as parameters in a system of equations to solve for each branch length in the consensus gene tree described above (Figure 8d). All Ks values for all species pairwise comparisons are given in Data S10.

### Divergence time estimation

The estimation of divergence times was conducted using BEAST2 (Bouckaert et al., 2014) on an alignment comprising 15 taxa. The data partition was modeled using a gamma site model with the WAG substitution model and gamma rate heterogeneity, employing four categories and a gamma shape parameter exponentially distributed with a mean of 1. A relaxed clock model was optimized for the partition, with the clock rate uniformly distributed between 0 and infinity. The phylogenetic tree was inferred using a Yule speciation model, with the birth rate uniformly distributed between 0 and infinity. Constraints on divergence times were incorporated through the implementation of maximum likelihood‐based coalescent‐constrained (MRCA) priors. The ingroup was assigned a normally distributed prior with a mean of 63.5 Mya (Koenen et al., 2021) and a *σ* of 1.0, while the outgroup (*Arabidopsis* and *Vitis*) was assigned a normally distributed prior with a mean of 100.0 Mya (Koenen et al., 2021) and *σ* of 1.0. The fossil calibration data utilized in this study is derived from the information presented in Koenen et al. (2021). The Markov‐chain Monte Carlo (MCMC) chain length was set to approximately 12 million iterations, and the effective sample sizes (ESS) were set to exceed 200. (We note that our time inferences would have differed depending on choice of priors; Zhao et al. (2021) reported an age of 67.3 Mya for the legume crow node, while Magallón et al. (2015) reported a much older age of 93.2 Mya).

### Analysis of gene family expansions and contractions and gene ontology enrichment

Homologous gene clustering was performed with the OrthoFinder (Emms & Kelly, 2019) clustering algorithm and default options (e‐value 1e‐2, inflation value 1.5) on the Orthovenn3 web server (Sun et al., 2023). Gene family contraction and expansion analysis was performed using CAFE5 (Mendes et al., 2020). Gene ontology (GO) terms for biological process, molecular function, and cellular component categories and enrichment of the expanded and contracted genes were then obtained. The enriched horizontal bar plot was drawn by using SRplot (Tang et al., 2023).

### Identification of centromeric arrays in *Cercis* and *Chamaecrista*

Tandem repeats in *C. canadensis*, *C. chinensis*, and *Ch. fasciculata* were identified using ULTRA (Olson & Wheeler, 2024). The most abundant repeats with length greater than 90 bases were evaluated for chromosomal position and array size. Based on these characteristics, putative centromeric repeats were selected and clustered to identify consensus centromeric repeat sequences (Methods and Results S1). The predominant satellite repeats in *C. canadensis* and *C. chinensis* were both 104 bases in length (though only 85.6% similar). In *C. fasciculata*, the predominant repeats were 351, 352, and 355 bases long. These sequences were used as BLAST queries against the *Cercis* and *Chamaecrista* genomes. For the purpose of identifying proportions of the genome with significant proportions of centromeric satellite repeats, BLAST hit locations were merged using the bedtools ‘merge’ command (Quinlan & Hall, 2010), with −*d* = 25 000 to allow merger hits within 25 kb of one another.

## CONFLICT OF INTEREST

The authors declare no conflict of interest.

## AUTHOR CONTRIBUTIONS

HL, JSS, and SBC conducted primary analyses, drafted the review, managed project data, and conducted gene family, phylogenomic, and Ks analyses. SBC and JSS collected plant tissue for genome sequencing. BDJ contributed analysis of genomic repeats. JG, JS, JJ, and RW conducted the genome sequencing and assembly. MW, JW, KK, and JE conducted lab work for genome sequencing and annotation. TB and SS generated genome annotations. DG and KB managed genome assembly and annotation work. QX, TH, PS, RB, AL, DS, and LJ carried out synteny analyses and contributed software for genomic analysis. JHL‐M and LTL reviewed the analyses and edited the manuscript. SBC and JL‐M conceptualized and designed the research. DMG, KB, and JHL‐M provided project management and funding. All authors approved the final draft.

## Supporting information


**Data S1.** Methods and results.

**Figure S1.** Syntenic dot plots of the *C. canadensis* and *Ch. fasciculata* genomes. Upper left seven chromosomes: *Cercis* self‐comparison. Lower right eight chromosomes: *Chamaecrista* genomes. Upper right quadrant: *Cercis x Chamaecrista*. Lower right quadrant: *Chamaecrista x Cercis*. Plot were generated by MCScanX (Wang et al., 2012), based on blastp matches with E‐value cutoff of 1e‐10, with mscanx MATCH_SIZE (min genes) set to 5. The output of the MCScanX utility ‘dissect_multiple_alignment’ is shown below. Cells in bold show the last gene count category with high value, indicating a duplication depth affecting a large proportion of genes in the respective comparison. For the self‐genome comparisons, these numbers indicate depths of 2 for *Cercis* and 5 for *Chamaecrista*, consistent with the hypothesized WGD histories: the gamma triplication evident in both, and an additional WGD near the base of the Caesalpinioideae affecting *Chamaecrista*. In each case, the numbers should be one fewer than the depth predicted by the WGD history, because the self‐comparisons show the number of syntenic homoeologs ‘seen’ by any given gene, not including the gene itself, i.e., 3 − 1 = 2 and (3 × 2) − 1 = 5. The main diagonal in the self‐comparisons is not counted. For the cross‐genome comparisons, the high synteny‐depth values indicate 6 for *Cercis* against *Chamaecrista*, and 3 for *Chamaecrista* against *Cercis*. These are also consistent with the hypothesized WGD histories: 3 and 3 × 2 = 6.
**Figure S2.** Syntenic dot plots of the *C. canadensis* (x‐axis) vs. *C. chinensis* (y‐axis). There are some mismatched regions, and inversions have occurred on chromosomes 3 and 5. Dots represent amino acid matches, as determined by MUMmer4 (Marçais et al., 2018).
**Figure S3.** Gene ontology (GO) enrichment analysis of *C. canadensis* by three functional groups. (A) biological processes (B) cellular components (C) molecular function.
**Figure S4.** Gene ontology (GO) enrichment analysis of *Ch. fasciculata* by three functional groups. (A) biological processes (B) cellular components (C) molecular function.
**Figure S5.** GO enrichment analysis in the comparison between *C. canadensis* and *B. variegata*. The top 30 terms with *P*‐value of −log10 were shown. The ontology categories are divided into three; BP: biological process, CC: cellular component, MF: molecular function. (A) represents the increased GO terms (B) represents the decreased GO terms.
**Figure S6.** GO enrichment analysis in the comparison between *Ch. fasciculata* and *S. tora*. The top 30 terms with *P*‐value of −log10 were shown. The ontology categories are divided into three; BP: biological process, CC: cellular component, MF: molecular function. (A) represents the increased GO terms (B) represents the decreased GO terms.
**Figure S7.** Density diagram by repeat class in *C. canadensis*. Since density is different for each repeat class, it is shown separately on the right legends.
**Figure S8.** Density diagram by repeat class in *Ch. fasciculata*. Since density is different for each repeat class, it is shown separately on the right legends.
**Figure S9.** Comparisons of haplotype assemblies for *C. canadensis*. Regions of higher densities (lighter) show higher identities, indicating either conserved synteny or repetitive sequence.
**Figure S10.** Comparisons of haplotype assemblies for *Ch. fasciculata*. Regions of higher densities (lighter) show higher identities, indicating either conserved synteny or repetitive sequence.
**Figure S11.** Dot plot of *Ch. fasciculata* (vertical) by *C. canadensis* (horizontal). Dots represent amino acid matches, determined by MUMmer4 (Marçais et al., 2018).

**Table S1.** List of genomes used in the initial gene family. For genera with multiple species, these were incorporated into genus‐level pangenes.
**Table S2.** Classification of repetitive elements of *C. canadensis* and *Ch. fasciculata*.
**Table S3.** Whole‐genome duplication and speciation peaks analysis by Ks value.

**Data S4.** Manuscript supplement; Compressed directory of aligned sequences from 2212 low‐copy gene families, for calculation of a consensus species tree.

**Data S5.** Manuscript supplement; Spreadsheet of gene families enriched for whole‐genome duplication signals.

**Data S6.** Manuscript supplement; Compressed directory of aligned sequences from 250 gene families with high rates of gene duplication consistent with whole‐genome duplications in the legume subfamilies.

**Data S7.** Manuscript supplement; Spreadsheet of gene families 36 gene families with members deriving from syntenic blocks from selected species.

**Data S8.** Manuscript supplement; Compressed directory of aligned sequences from 36 gene families with members deriving from syntenic blocks from selected species.

**Data S9.** Manuscript supplement; Spreadsheet with scorings of gene families for phylogenetic patterns consistent with either allo‐ or auto‐polyploidy in the Papilionoid and Caesalpinioid subfamilies.

**Data S10.** Manuscript supplement; Spreadsheet of Ks plots for all species pairs.

## Data Availability

BioProject for *Chamaecrista fasciculata* var. ISC494698: https://www.ncbi.nlm.nih.gov/bioproject/PRJNA1137390. BioProject for *Cercis canadensis* ISC453364: https://www.ncbi.nlm.nih.gov/bioproject/PRJNA1137384. Genome assemblies and annotations for *Chamaecrista fasciculata* var. ISC494698 and *Cercis canadensis* ISC453364: https://phytozome‐next.jgi.doe.gov. Legume gene families and associated phylogenomic analyses: https://data.legumeinfo.org/LEGUMES/Fabaceae/genefamilies/legume.fam3.VLMQ/.
