# Supplementary material for: Legume genome structures and histories inferred from Cercis canadensis and Chamaecrista fasciculata genomes
Source: Plant J. 2026 Jun 10;126(5):e70981. doi: 10.1111/tpj.70981 (PMC13252982; doi:10.1111/tpj.70981)
Supplement: Supplementary file 2 — Figure S1. Syntenic dot plots of the C. canadensis and Ch. fasciculata genomes. Upper left seven chromosomes: Cercis self‐comparison. Lower right eight chromosomes: Chamaecrista genomes. Upper right quadrant: Cercis x Chamaecrista. Lower right quadrant: Chamaecrista x Cercis. Plot were generated by MCScanX (Wang et al., 2012), based on blastp matches with E‐value cutoff of 1e‐10, with mscanx MATCH_SIZE (min genes) set to 5. The output of the MCScanX utility ‘dissect_multiple_alignment’ is shown below. Cells in bold show the last gene count category with high value, indicating a duplication depth affecting a large proportion of genes in the respective comparison. For the self‐genome comparisons, these numbers indicate depths of 2 for Cercis and 5 for Chamaecrista, consistent with the hypothesized WGD histories: the gamma triplication evident in both, and an additional WGD near the base of the Caesalpinioideae affecting Chamaecrista. In each case, the numbers should be one fewer than the depth predicted by the WGD history, because the self‐comparisons show the number of syntenic homoeologs ‘seen’ by any given gene, not including the gene itself, i.e., 3 − 1 = 2 and (3 × 2) − 1 = 5. The main diagonal in the self‐comparisons is not counted. For the cross‐genome comparisons, the high synteny‐depth values indicate 6 for Cercis against Chamaecrista, and 3 for Chamaecrista against Cercis. These are also consistent with the hypothesized WGD histories: 3 and 3 × 2 = 6. Figure S2. Syntenic dot plots of the C. canadensis (x‐axis) vs. C. chinensis (y‐axis). There are some mismatched regions, and inversions have occurred on chromosomes 3 and 5. Dots represent amino acid matches, as determined by MUMmer4 (Marçais et al., 2018). Figure S3. Gene ontology (GO) enrichment analysis of C. canadensis by three functional groups. (A) biological processes (B) cellular components (C) molecular function. Figure S4. Gene ontology (GO) enrichment analysis of Ch. fasciculata by three functional [file TPJ-126-0-s008.docx]

**Supporting Information – Supplementary Figures for Article: Legume genome structures and histories inferred from *Cercis canadensis* and *Chamaecrista fasciculata* genomes**

**Authors** : Hyun-oh Lee, Jacob S Stai, Qiaoji Xu, Thulani Hewavithana, Rabnoor Batra, Alex Liu, Brandon D Jordan, Rachel Walstead, Jerry Jenkins, Melissa Williams, Jenell Webber, Jane Grimwood, John T Lovell, Tomáš Brůna, Shengqiang Shu, Keykhosrow Keymanesh, Joanne Eichenberger, Jeremy Schmutz, David M Goodstein, Kerrie Barry, David Sankoff, Lingling Jin, James H Leebens-Mack, Steven B Cannon

**Supplementary Figure S1.** Syntenic dotplots of the *C. canadensis* and *Ch. fasciculata* genomes. Upper left seven chromosomes: *Cercis* self-comparison. Lower right eight chromosomes: *Chamaecrista* genomes. Upper right quadrant: *Cercis x Chamaecrista*. Lower right quadrant: *Chamaecrista x Cercis*. Plot were generated by MCScanX (Wang, Tang et al., 2012), based on blastp matches with E-value cutoff of 1e-10, with mscanx MATCH_SIZE (min genes) set to 5. The output of the MCScanX utility “dissect_multiple_alignment” is shown below. Cells in bold show the last gene-count category with high value, indicating a duplication depth affecting a large proportion of genes in the respective comparison. For the self-genome comparisons, these numbers indicate depths of 2 for *Cercis* and 5 for *Chamaecrista*, consistent with the hypothesized WGD histories: the gamma triplication evident in both, and an additional WGD near the base of the Caesalpinioideae affecting *Chamaecrista.* In each case, the numbers should be one fewer than the depth predicted by the WGD history, because the self-comparisons show the number of syntenic homoeologs “seen” by any given gene, not including the gene itself, i.e., 3-1=2 and (3x2)-1=5. The main diagonal in the self-comparisons is not counted. For the cross-genome comparisons, the high synteny-depth values indicate 6 for *Cercis* against *Chamaecrista*, and 3 for *Chamaecrista* against *Cercis*. These are also consistent with the hypothesized WGD histories: 3 and 3x2=6.

| **Self-genome comparison:** | |  |  |  |  |  |  |  |  |
| --- | --- | --- | --- | --- | --- | --- | --- | --- | --- |
| Duplication depth | 0 | 1 | **2** | 3 | 4 | **5** | 6 | 7 | 8 |
| Reference: *Cercis* | 11558 | 7477 | **7738** | 169 | 14 | 8 | 24 | 21 | 2 |
| Reference: *Chamae* | 4213 | 9672 | 5529 | 4923 | 3205 | **1397** | 1 | 22 |  |
|  |  |  |  |  |  |  |  |  |  |
| **Cross-genome comparison:** | |  |  |  |  |  |  |  |  |
| Duplication depth | 0 | 1 | 2 | **3** | 4 | 5 | **6** | 7 | 8 |
| Reference: *Chamae* | 1144 | 10721 | 9936 | **6885** | 269 | 7 |  |  |  |
| Reference: *Cercis* | 2975 | 3567 | 6821 | 4020 | 4602 | 3411 | **1612** | 162 | 3 |


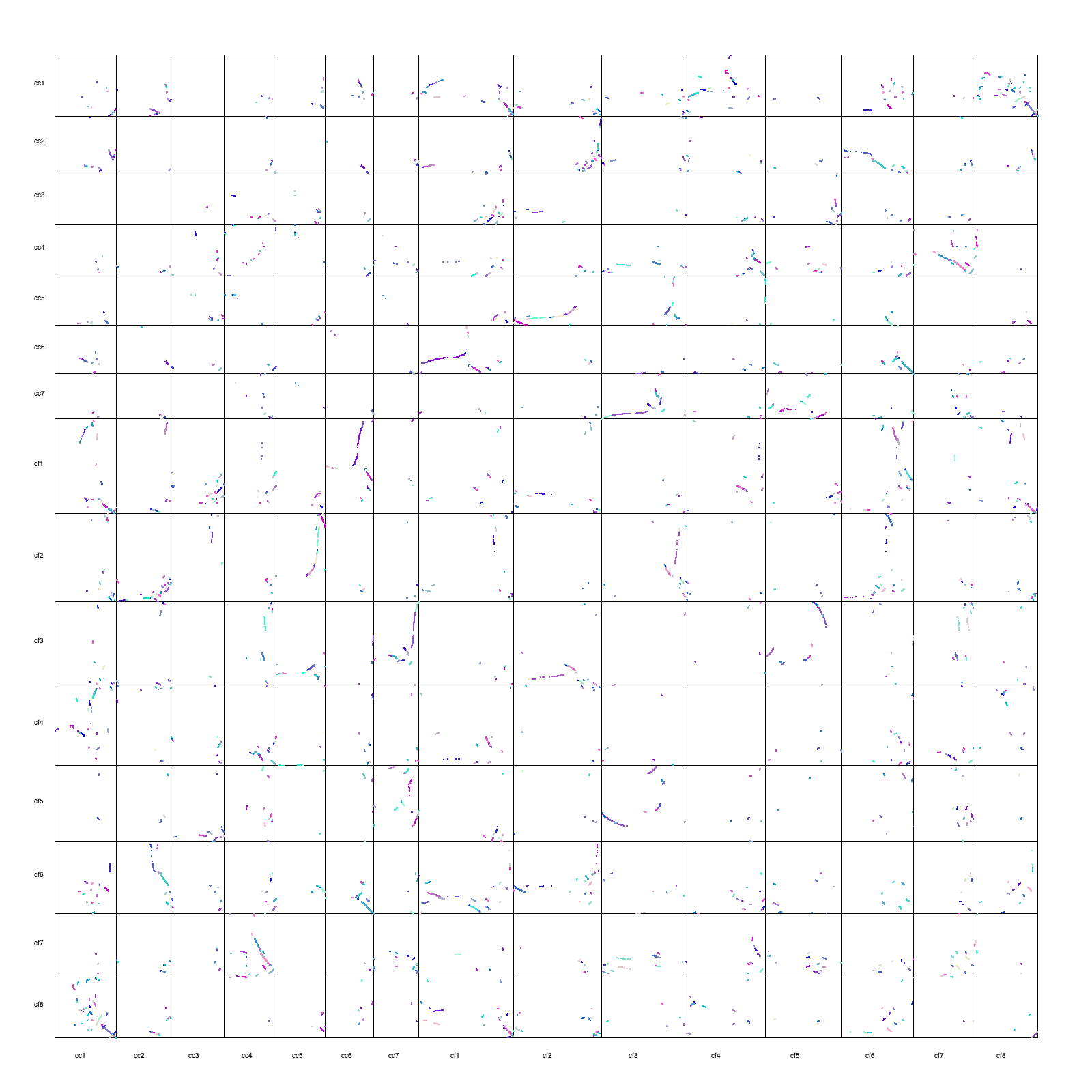


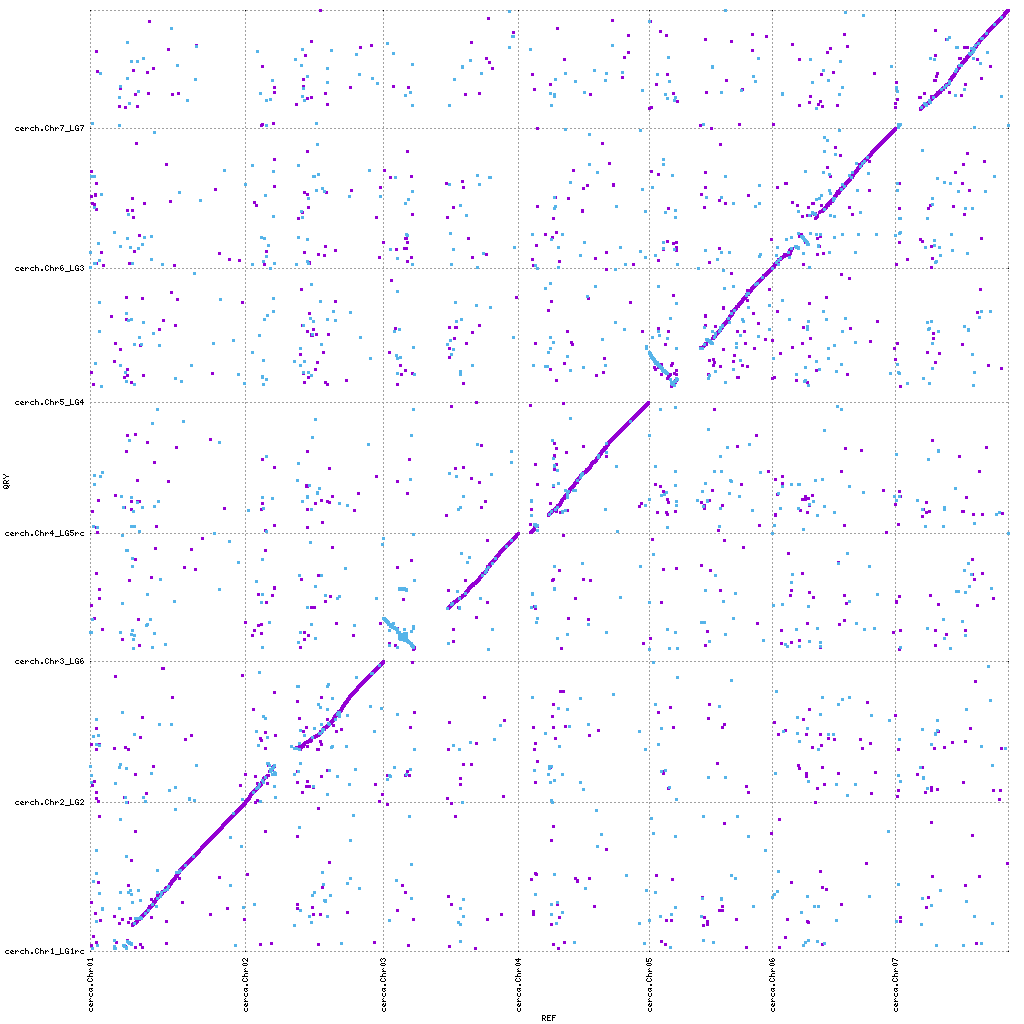


**Supplementary Figure S2.** Syntenic dotplots of the *C. canadensis* (x-axis) vs. *C. chinensis* (y-axis)*.* There are some mismatched regions, and inversions have occurred on chromosomes 3 and 5. Dots represent amino acid matches, as determined by MUMmer4 (Marçais et al., 2018).

**
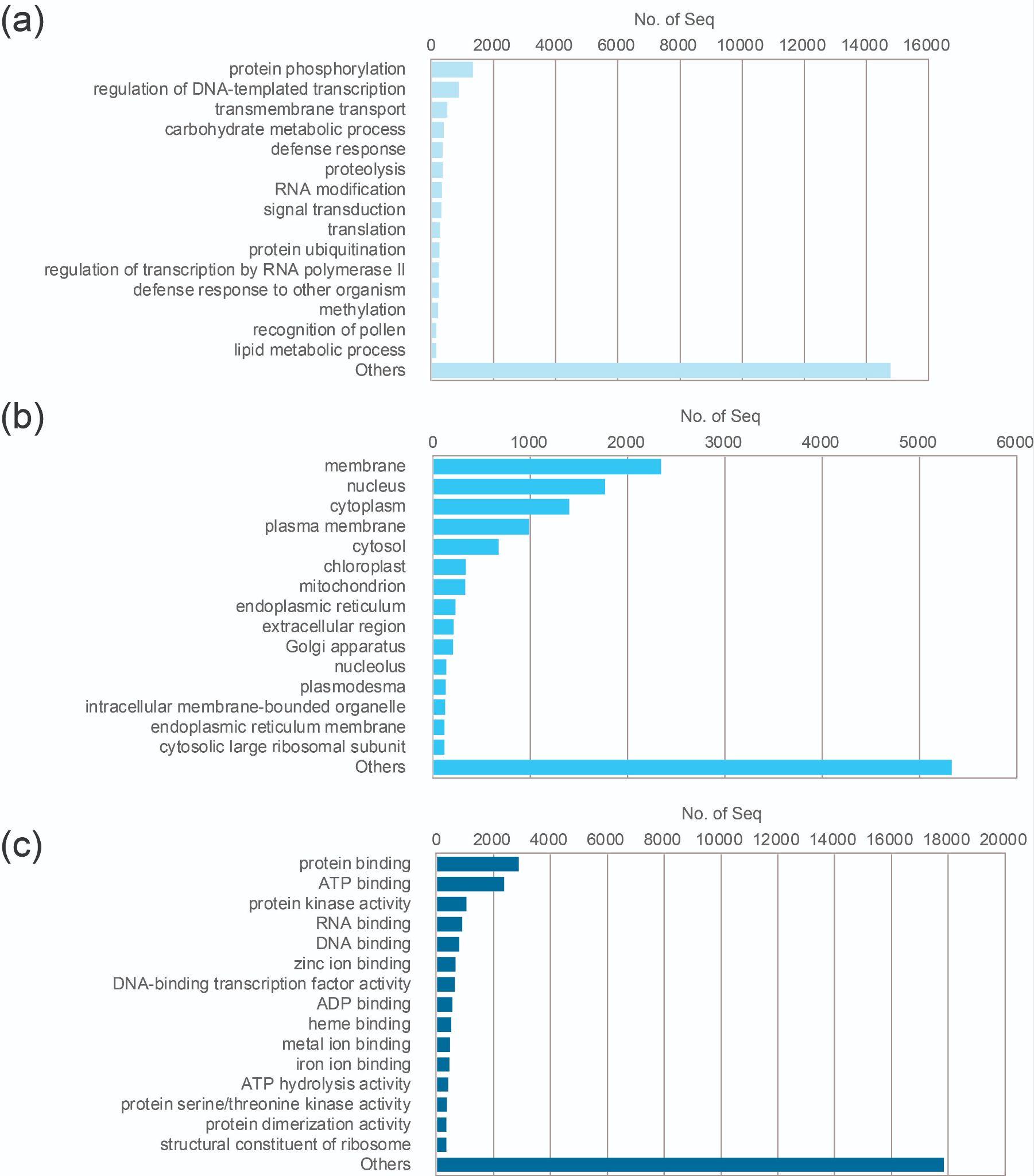
**

**Supplementary Figure S3**. Gene ontology (GO) enrichment analysis of *C. canadensis* by three functional groups. (A) biological processes (B) cellular components (C) molecular function


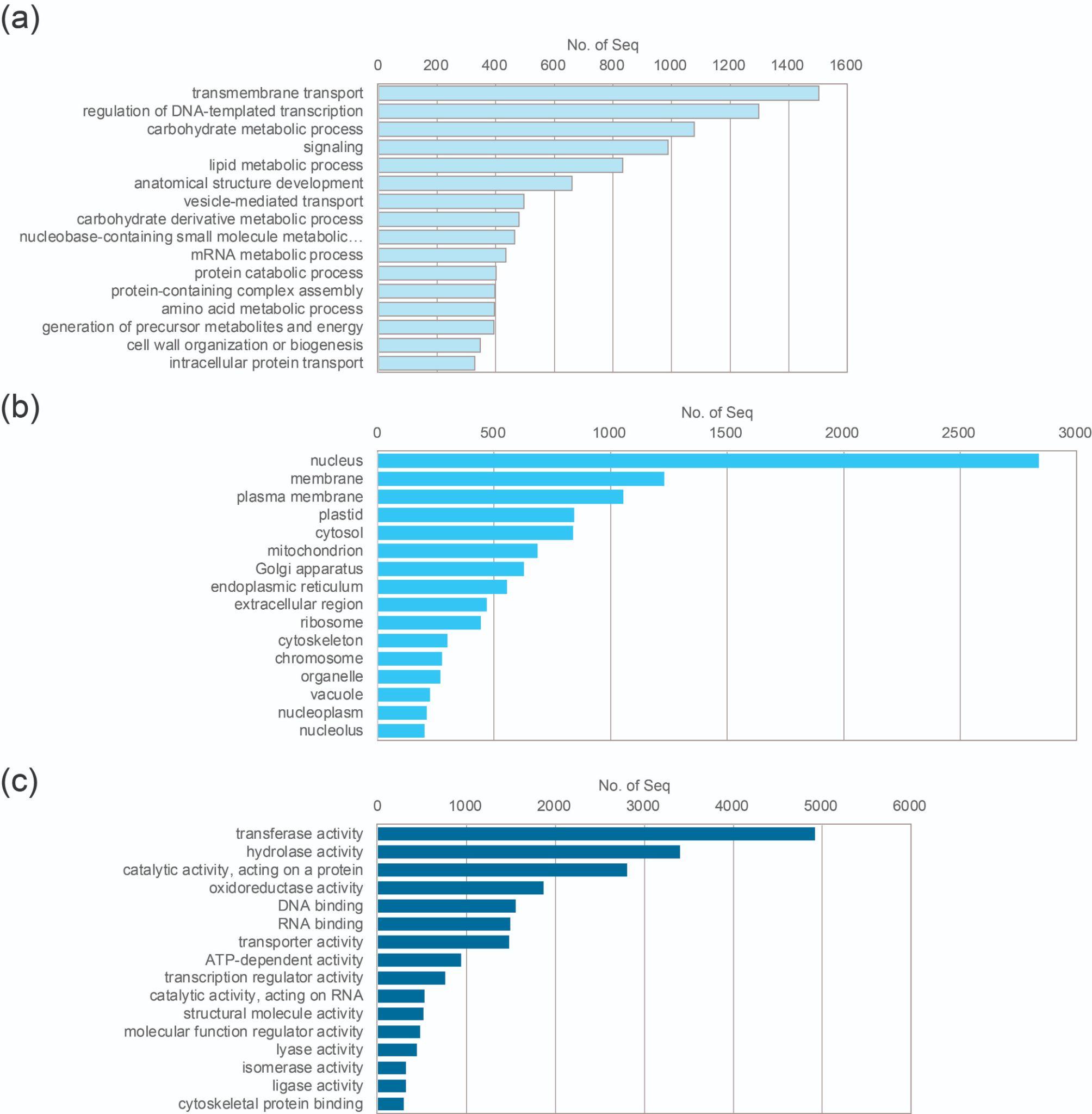


**Supplementary Figure S4**. Gene ontology (GO) enrichment analysis of *C. fasciculata* by three functional groups. (A) biological processes (B) cellular components (C) molecular function


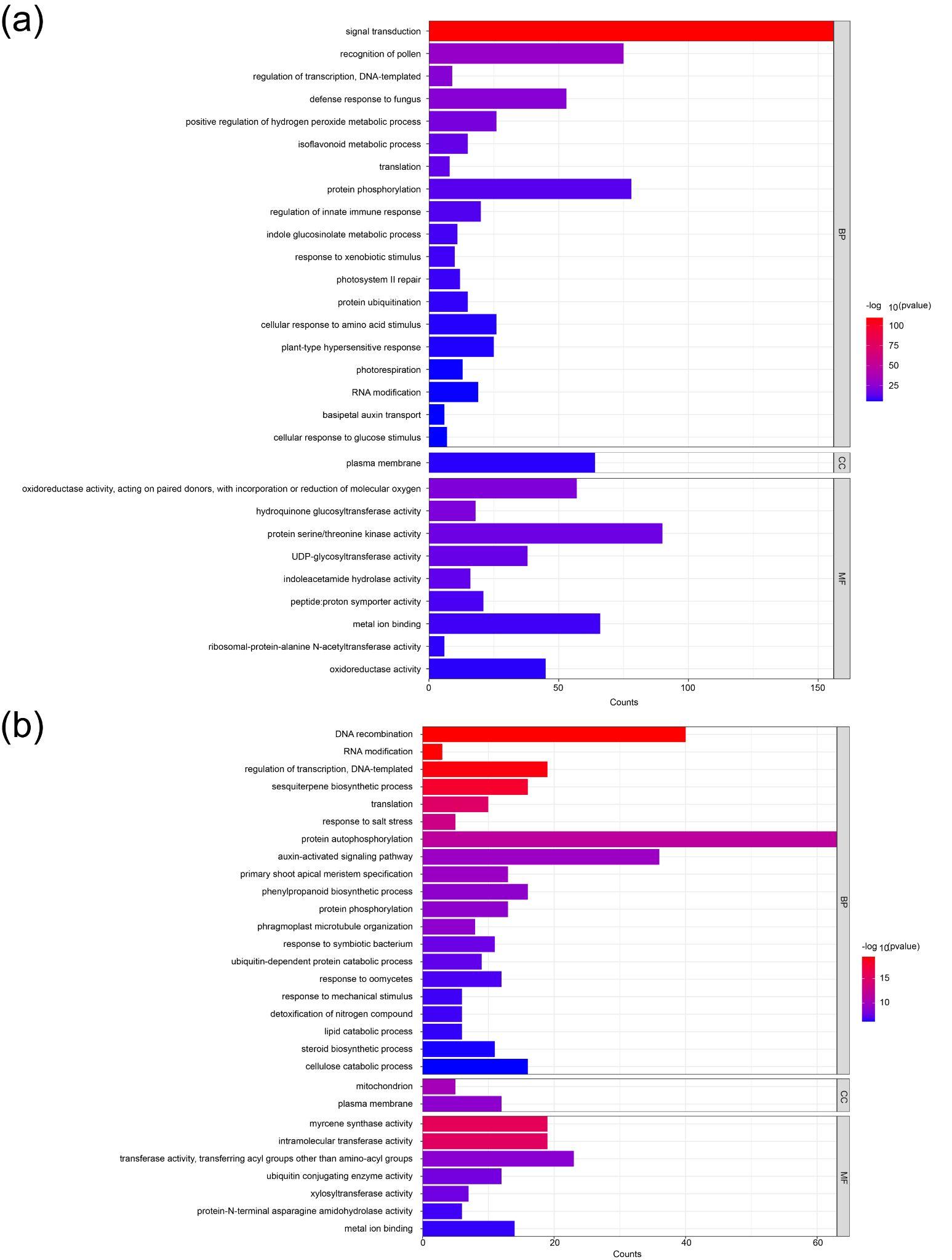


**Supplementary Figure S5.** GO enrichment analysis in the comparison between *C. canadensis* and *B. variegata*. The top 30 terms with p-value of -log10 were shown. The ontology categories are divided into three; BP: biological process, CC: cellular component, MF: molecular function. (A) represents the increased GO terms (B) represents the decreased GO terms.


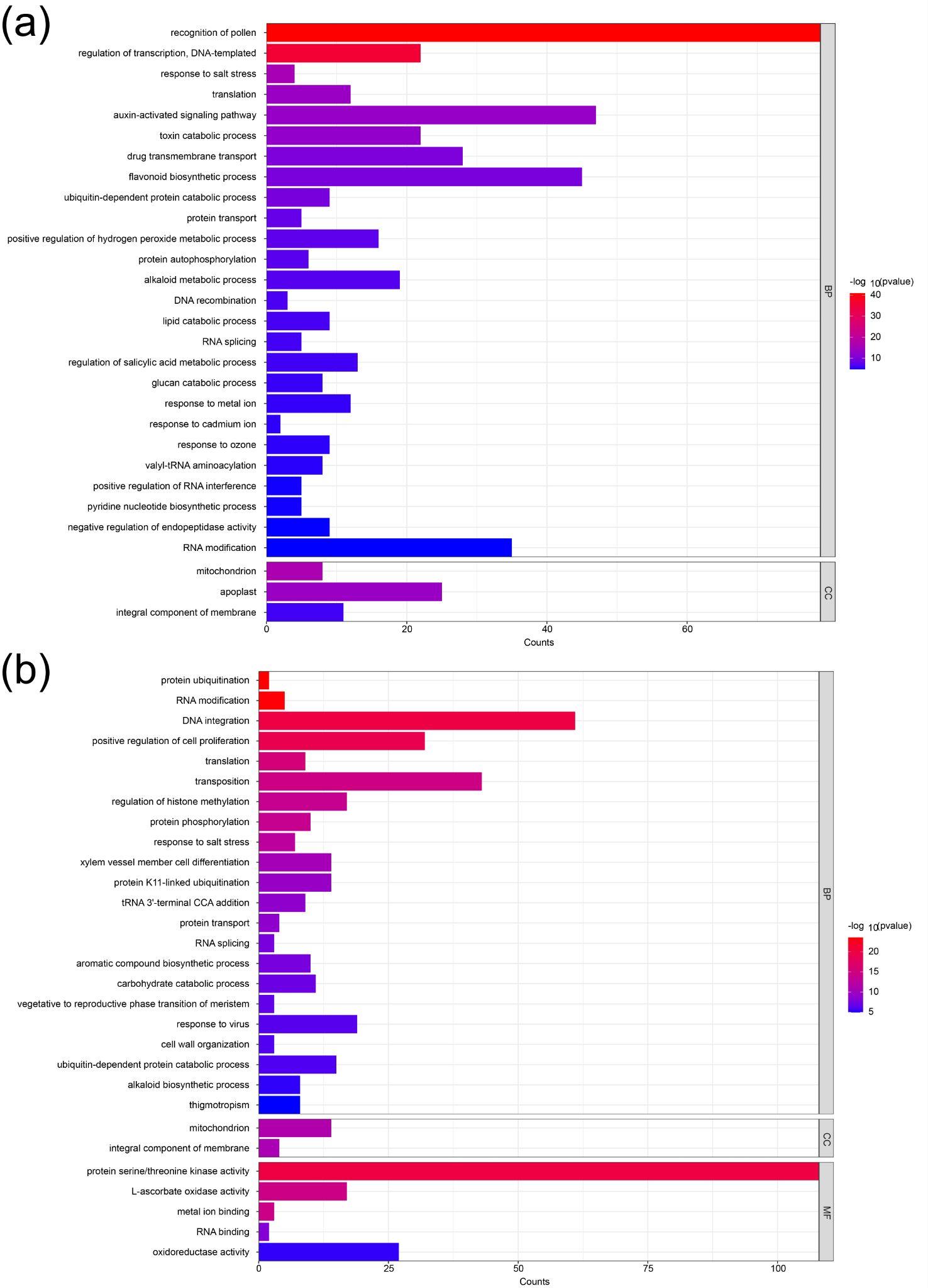


**Supplementary Figure S6.** GO enrichment analysis in the comparison between *C. fasciculata* and *S. tora*. The top 30 terms with p-value of -log10 were shown. The ontology categories are divided into three; BP: biological process, CC: cellular component, MF: molecular function. (A) represents the increased GO terms (B) represents the decreased GO terms.

**
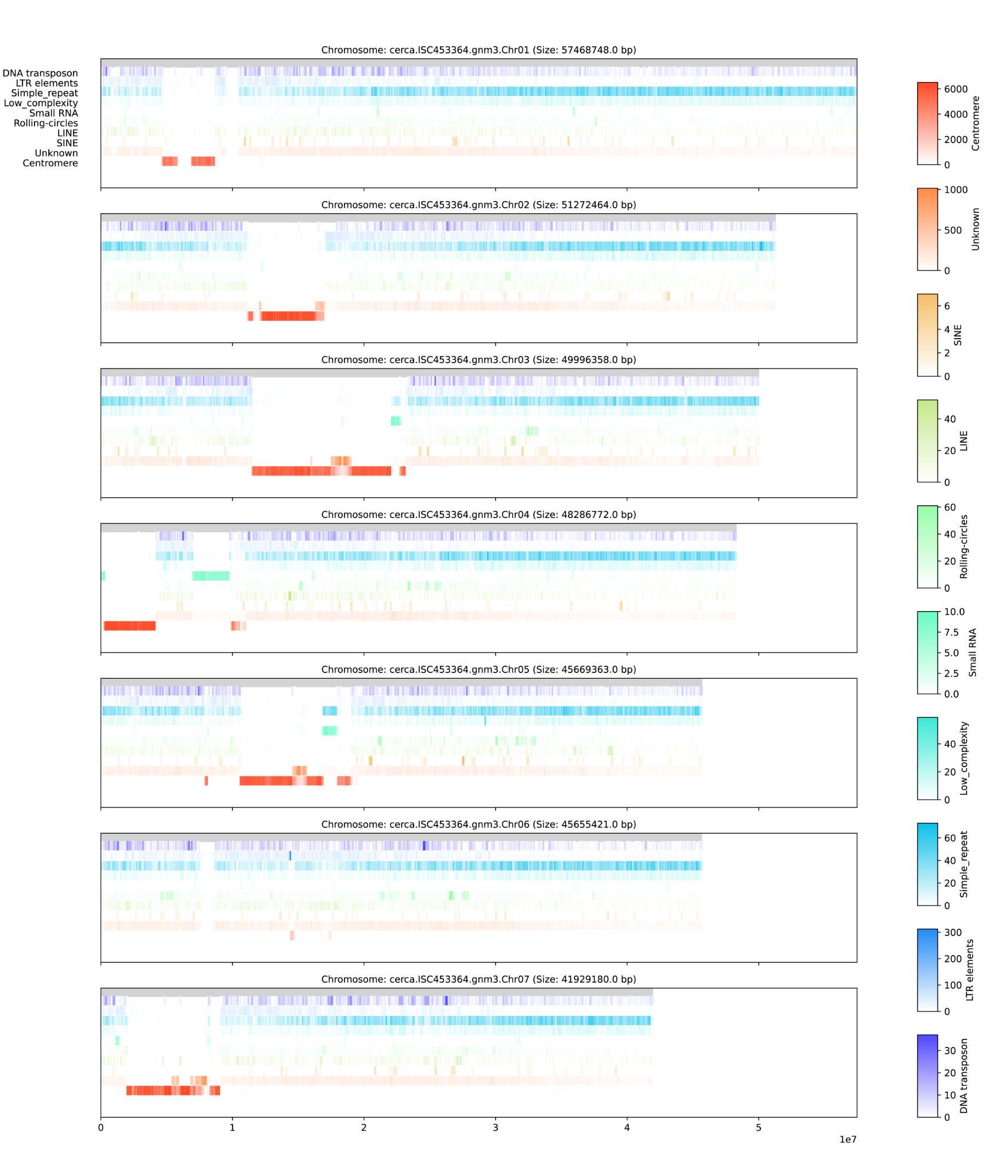
**

**Supplementary Figure S7.** Density diagram by repeat class in *C. canadensis*. Since density is different for each repeat class, it is shown separately on the right legends.

**
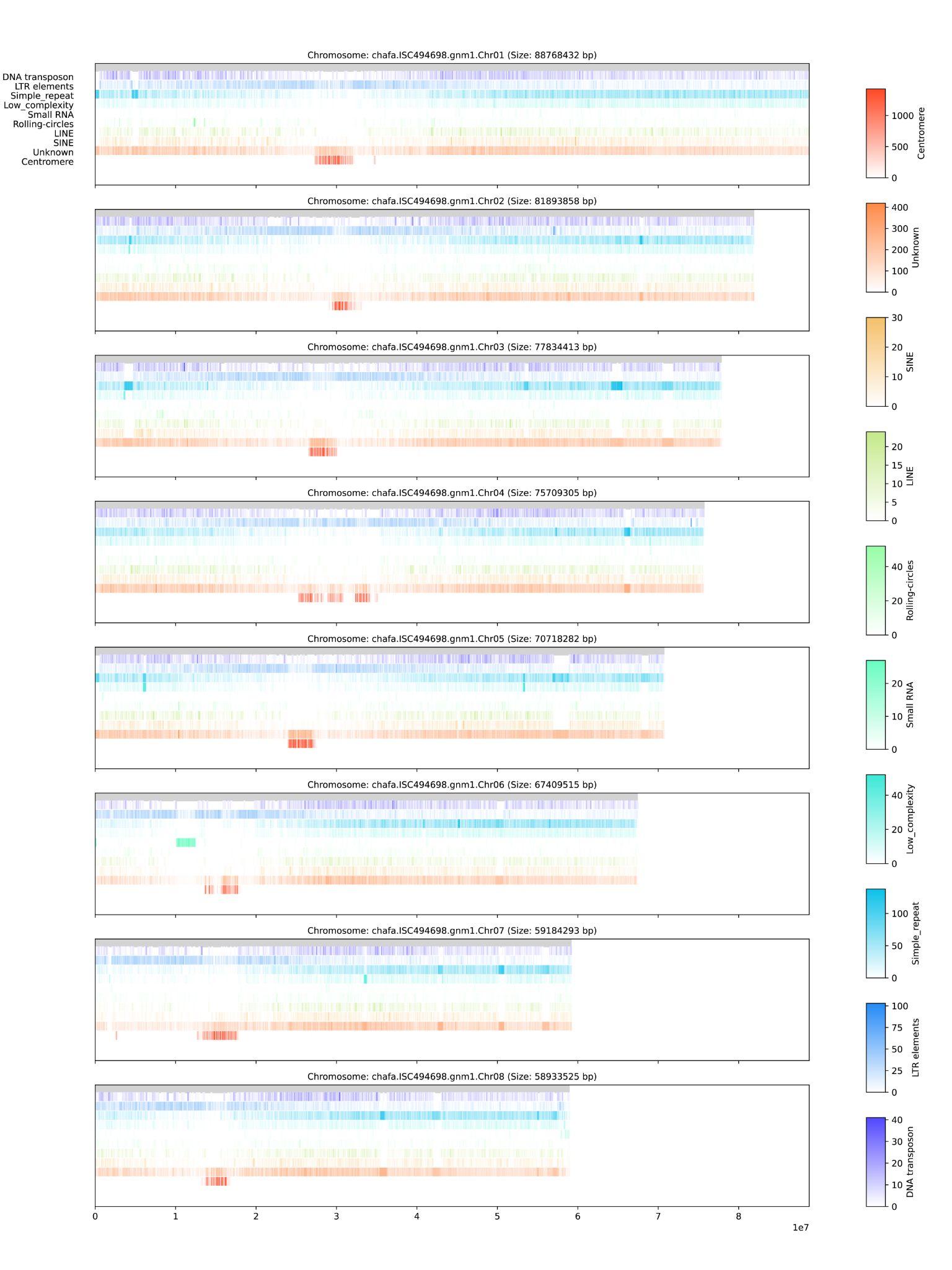
**

**Supplementary Figure S8.** Density diagram by repeat class in *Ch. fasciculata*. Since density is different for each repeat class, it is shown separately on the right legends.


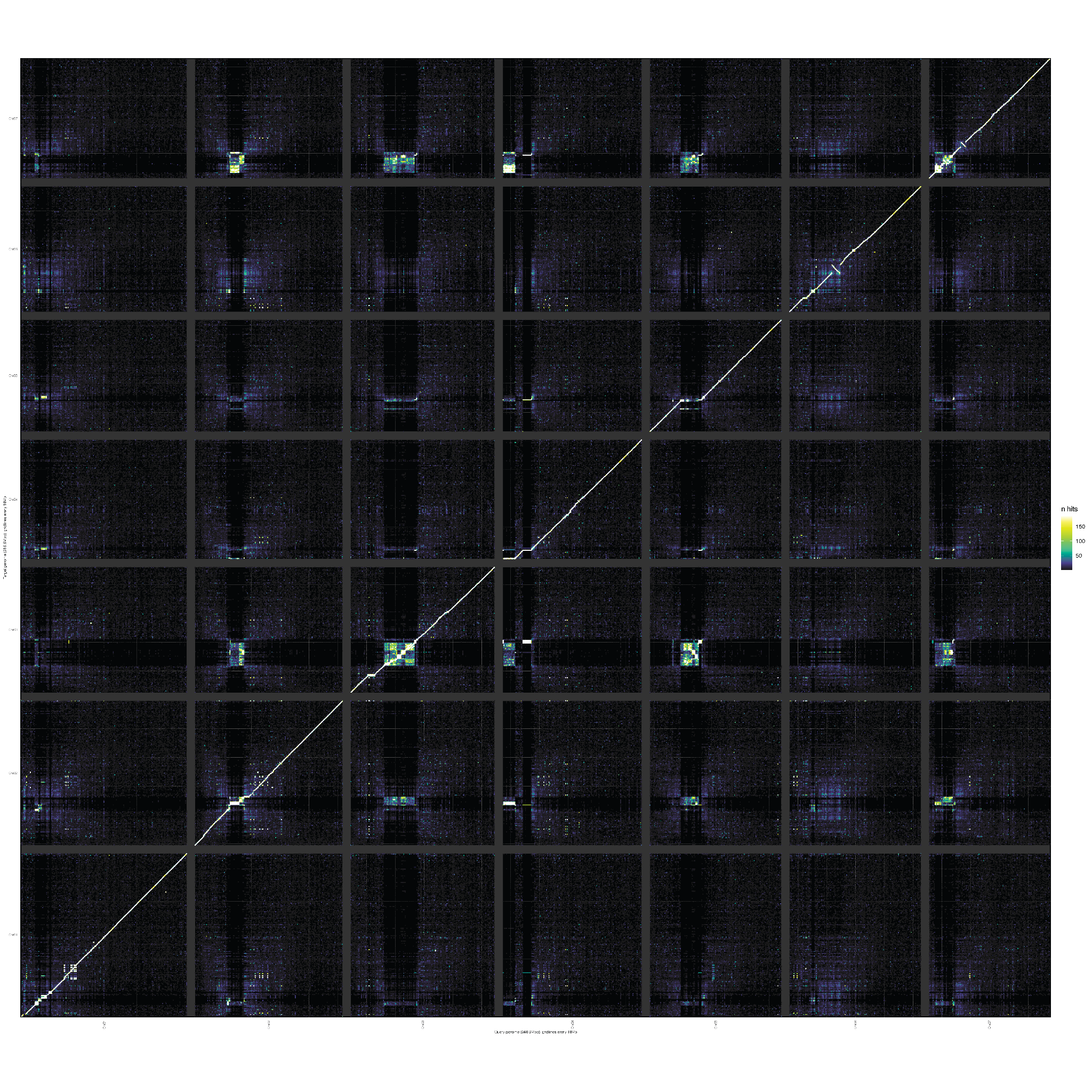


**Supplementary Figure S9.** Comparisons of haplotype assemblies for *C. canadensis*. Regions of higher densities (lighter) show higher identities, indicating either conserved synteny or repetitive sequence.


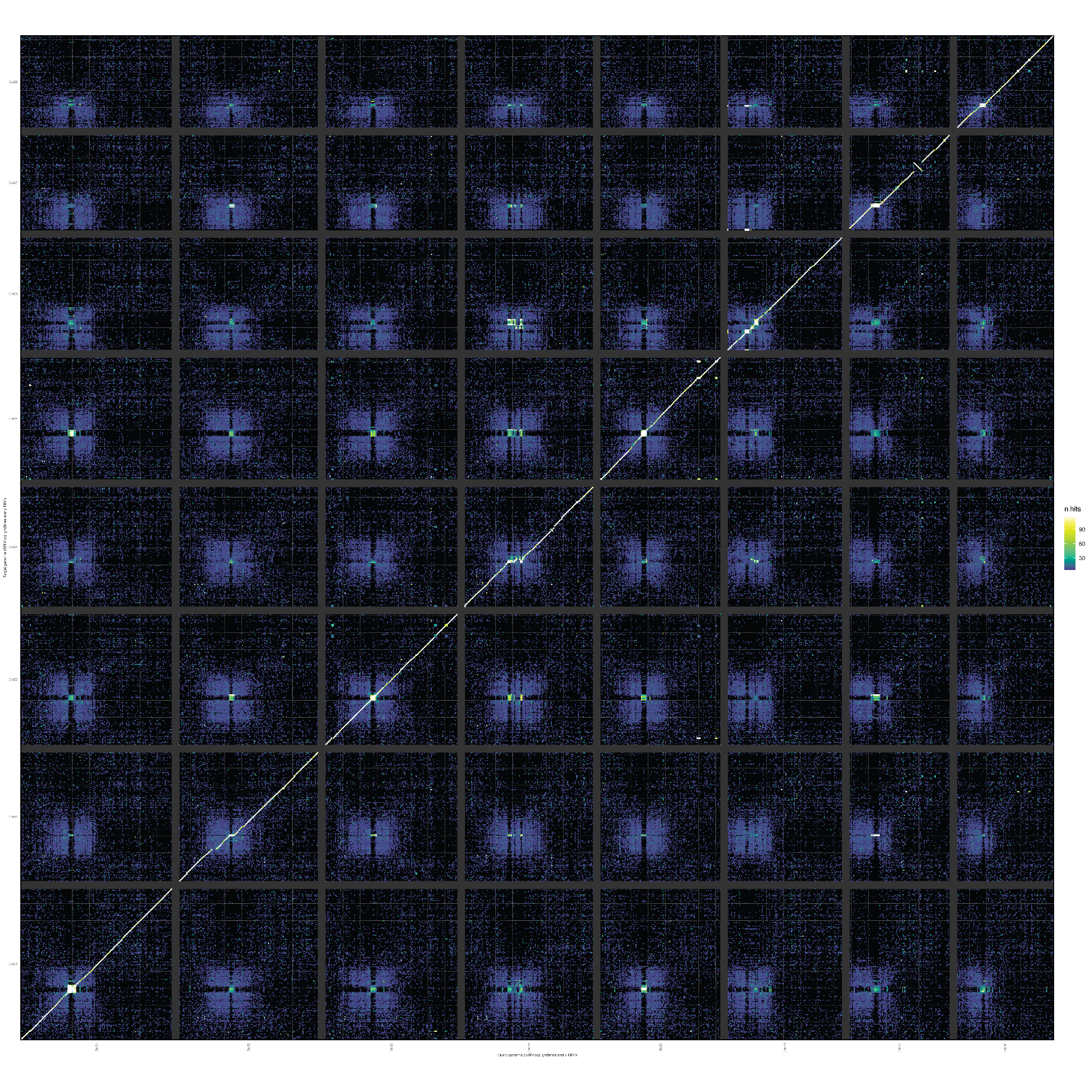


**Supplementary Figure S10.** Comparisons of haplotype assemblies for *Ch. fasciculata*. Regions of higher densities (lighter) show higher identities, indicating either conserved synteny or repetitive sequence.


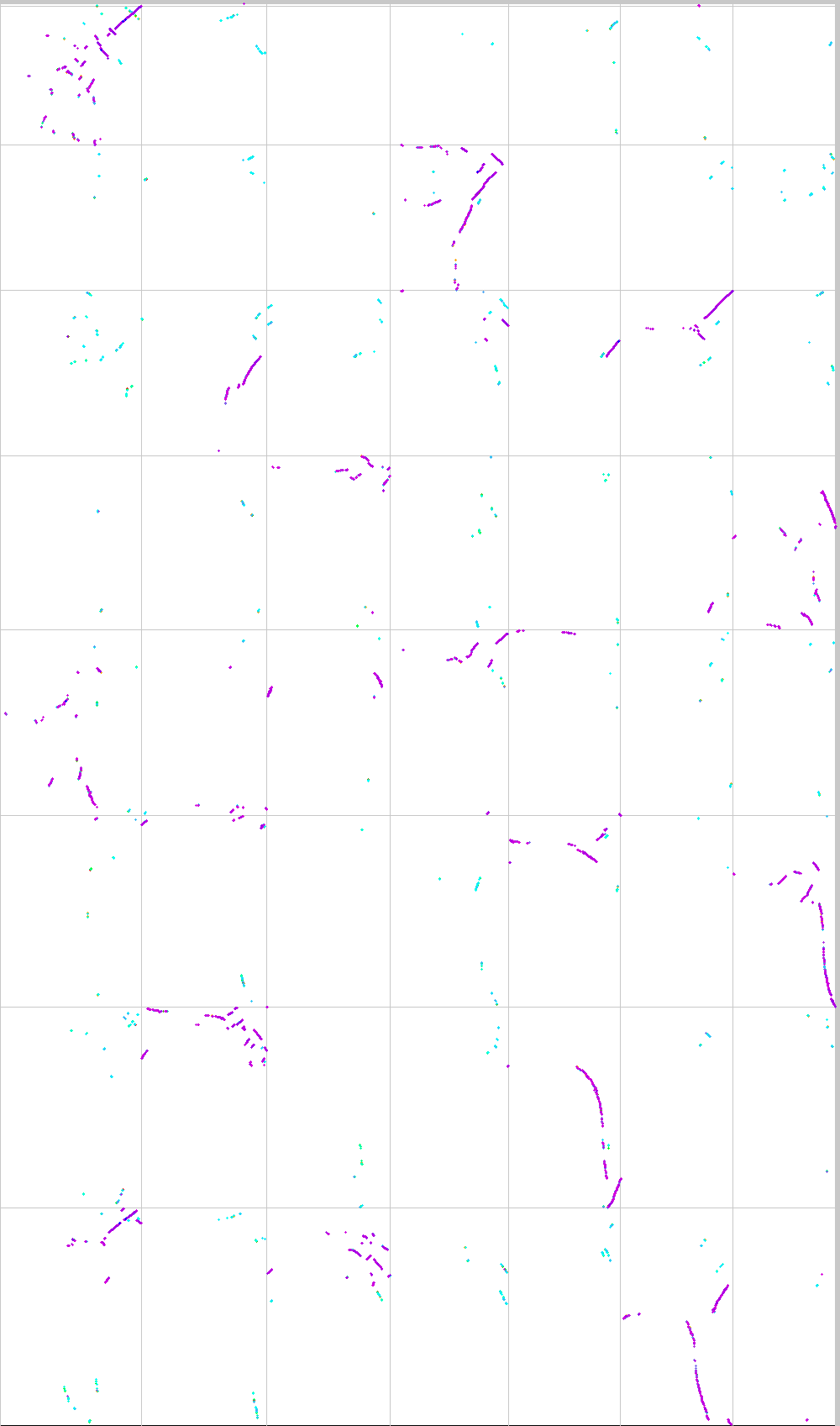


**Supplementary Figure S11.** Dot plot of *Ch. fasciculata* (vertical) by *C. canadensis* (horizontal). Dots represent amino acid matches, determined by MUMmer4 (Marçais et al., 2018).
