# Supplementary material for: Legume genome structures and histories inferred from Cercis canadensis and Chamaecrista fasciculata genomes
Source: Plant J. 2026 Jun 10;126(5):e70981. doi: 10.1111/tpj.70981 (PMC13252982; doi:10.1111/tpj.70981)
Supplement: Supplementary file 3 — Table S1. List of genomes used in the initial gene family. For genera with multiple species, these were incorporated into genus‐level pangenes. Table S2. Classification of repetitive elements of C. canadensis and Ch. fasciculata. Table S3. Whole‐genome duplication and speciation peaks analysis by Ks value. [file TPJ-126-0-s007.docx]

**Supporting Information – Supplementary Tables for Article: Legume genome structures and histories inferred from *Cercis canadensis* and *Chamaecrista fasciculata* genomes**

**Authors** : Hyun-oh Lee, Jacob S Stai, Qiaoji Xu, Thulani Hewavithana, Rabnoor Batra, Alex Liu, Brandon D Jordan, Rachel Walstead, Jerry Jenkins, Melissa Williams, Jenell Webber, Jane Grimwood, John T Lovell, Tomáš Brůna, Shengqiang Shu, Keykhosrow Keymanesh, Joanne Eichenberger, Jeremy Schmutz, David M Goodstein, Kerrie Barry, David Sankoff, Lingling Jin, James H Leebens-Mack, Steven B Cannon

**Supplementary Table S1.** List of genomes used in the initial gene family. For genera with multple species, these were incorporated into genus-level pangenes.

| **Genus** | **Species** | **Subfamily** | **Citation** | **doi** |
| --- | --- | --- | --- | --- |
| ***Acacia*** | *crassocarpa* | Caesalpinioideae | Massaro et al., 2023 | 10.1093/g3journal/jkad284 |
| ***Aeschynomene*** | *evenia* | Papilionoideae | Quilbé et al., 2021 | 10.1038/s41467-021-21094-7 |
| ***Cercis*** | *canadensis* | Cercidoideae | **This manuscript** |  |
| ***Chamaecrista*** | *fasciculata* | Caesalpinioideae | **This manuscript** |  |
| ***Dalbergia*** | *odorata* | Papilionoideae | Hong et al., 2020 | 10.1093/gigascience/giaa084 |
| ***Lablab*** | *purpureus* | Papilionoideae | Njaci et al., 2023 | 10.1038/s41467-023-37489-7 |
| ***Lens*** | *culinaris* | Papilionoideae | Ramsay et al., 2021 | 10.1101/2021.07.23.453237 |
| ***Lotus*** | *japonicus* | Papilionoideae | Sato et al., 2008 | 10.1093/dnares/dsn008 |
| ***Lupinus*** | *albus* | Papilionoideae | Hufnagel et al., 2020 | 10.1038/s41467-019-14197-9 |
| ***Phanera*** | *championii* | Cercidoideae | Lu et al., 2024 | 10.1111/tpj.16620 |
| ***Pisum*** | *sativum* | Papilionoideae | Kreplak et al., 2019 | 10.1038/s41588-019-0480-1 |
| ***Senna*** | *tomentosa* | Papilionoideae | Kang et al., 2020 | 10.1038/s41467-020-19681-1 |
| ***Sindora*** | *glauca* | Detarioideae | Yu et al., 2022 | 10.3389/fpls.2021.794830 |
| ***Trifolium*** | *pratense* | Papilionoideae | De Vega et al., 2015 | 10.1038/srep17394 |
| ***Vicia*** | *faba* | Papilionoideae | Jayakodi et al., 2023 | 10.1038/s41586-023-05791-5 |
| ***Arachis*** | *hypogaea* | Papilionoideae | Bertioli et al., 2019 | 10.1038/s41588-019-0405-z |
| ***Arachis*** | *stenosperma* | Papilionoideae | Bertioli et al., 2019 | 10.1038/s41588-019-0405-z |
| ***Arachis*** | *duranensis* | Papilionoideae | Bertioli et al., 2016 | 10.1038/ng.3517 |
| ***Arachis*** | *ipaensis* | Papilionoideae | Bertioli et al., 2016 | 10.1038/ng.3517 |
| ***Cicer*** | *arietinum* | Papilionoideae | Garg et al., 2021 | 10.1016/j.jare.2021.10.009 |
| ***Cicer*** | *echinospermum* | Papilionoideae | Cook et al., 2022 | GenBank GCA_002896215.2 |
| ***Cicer*** | *reticulatum* | Papilionoideae | Cook et al., 2022 | GenBank GCA_002896235.1 |
| ***Glycine*** | *max* | Papilionoideae | Schmutz et al., 2010 | 10.1038/nature08670 |
| ***Glycine*** | *soja* | Papilionoideae | Xie et al., 2019 | 10.1038/s41467-019-09142-9 |
| ***Glycine*** | *cyrtoloba* | Papilionoideae | Zhuang et al., 2022 | 10.1038/s41477-022-01102-4 |
| ***Glycine*** | *dolichocarpaD3* | Papilionoideae | Zhuang et al., 2022 | 10.1038/s41477-022-01102-4 |
| ***Glycine*** | *tomentella-D3* | Papilionoideae | Zhuang et al., 2022 | 10.1038/s41477-022-01102-4 |
| ***Glycine*** | *falcata* | Papilionoideae | Zhuang et al., 2022 | 10.1038/s41477-022-01102-4 |
| ***Glycine*** | *stenophita* | Papilionoideae | Zhuang et al., 2022 | 10.1038/s41477-022-01102-4 |
| ***Glycine*** | *syndetika* | Papilionoideae | Zhuang et al., 2022 | 10.1038/s41477-022-01102-4 |
| ***Medicago*** | *truncatula* | Papilionoideae | Tang et al., 2014 | 10.1186/1471-2164-15-312 |
| ***Medicago*** | *sativa* | Papilionoideae | Chen et al., 2020 | 10.1038/s41467-020-16338-x |
| ***Phaseolus*** | *acutifolius* | Papilionoideae | Moghaddam et al., 2021 | 10.1038/s41467-021-22858-x |
| ***Phaseolus*** | *lunatus* | Papilionoideae | Garcia et al., 2021 | 10.1038/s41467-021-20921-1 |
| ***Phaseolus*** | *vulgaris* | Papilionoideae | Schmutz et al., 2014 | 10.1038/ng.3008 |
| ***Vigna*** | *angularis* | Papilionoideae | Sakai et al., 2015 | 10.1038/srep16780 |
| ***Vigna*** | *radiata* | Papilionoideae | Ha et al., 2021 | 10.1002/tpg2.20121 |
| ***Quillaja*** | *saponaria* | Quillajaceae | Reed et al., 2023 | 10.1126/science.adf3727 |
| ***Arabidopsis*** | *thaliana* | Brassicaceae | Cheng et al., 2017 | 10.1111/tpj.13415 |
| ***Prunus*** | *persica* | Rosaceae | Verde et al., 2017 | 10.1186/s12864-017-3606-9 |
| ***Vitis*** | *vinifera* | Vitaceae | The French–Italian Public Consortium for Grapevine Genome Characterization, 2007 | 10.1038/nature06148 |

**Supplementary Table S2.** Classification of repetitive elements of *C. canadensis* and *C. fasciculata*

|  |  | ***C. canadensis*** | | | ***C. fasciculata*** | | |
| --- | --- | --- | --- | --- | --- | --- | --- |
| **Type** |  | **Number of elements** | **Length occupied (bp)** | **Percentage of sequence (%)** | **Number of elements** | **Length occupied (bp)** | **Percentage of sequence (%)** |
| **Retroelements** |  | 46,144 | 44,016,712 | 12.94% | 98,544 | 167,631,216 | 28.88% |
| **SINEs** |  | 330 | 66,257 | 0.02% | 16,321 | 3,305,483 | 0.57% |
| **Penelope** |  | - | - | 0.00% | 3,345 | 536,935 | 0.09% |
| **LINEs** |  | 12,009 | 7,197,792 | 2.12% | 13,915 | 7,156,358 | 1.23% |
|  | CRE/SLACS | - | - | 0.00% | - | - | 0.00% |
|  | L2/CR1/Rex | - | - | 0.00% | 33 | 37,051 | 0.01% |
|  | R1/LOA/Jockey | 1,889 | 3,253,041 | 0.96% | - | - | 0.00% |
|  | R2/R4/NeSL | - | - | 0.00% | - | - | 0.00% |
|  | RTE/Bov-B | 1,716 | 269,684 | 0.08% | 2,258 | 464,649 | 0.08% |
|  | L1/CIN4 | 8,332 | 3,627,676 | 1.07% | 11,624 | 6,654,658 | 1.15% |
| **LTR elements** |  | 33,805 | 36,752,663 | 10.80% | 68,308 | 157,169,375 | 27.08% |
|  | BEL/Pao | 447 | 51,271 | 0.02% | - | - | 0.00% |
|  | Ty1/Copia | 14,850 | 11,910,975 | 3.50% | 51,586 | 133,215,406 | 22.95% |
|  | Gypsy/DIRS1 | 17,270 | 22,898,626 | 6.73% | 14,764 | 23,075,603 | 3.98% |
|  | Retroviral | 173 | 71,013 | 0.02% | 917 | 210,672 | 0.04% |
| **DNA transposons** |  | 14,340 | 7,213,145 | 2.12% | 30,546 | 8,727,862 | 1.50% |
|  | hobo-Activator | 5,152 | 1,702,409 | 0.50% | 4,617 | 1,214,584 | 0.21% |
|  | Tc1-IS630-Pogo | 124 | 92,957 | 0.03% | 10,632 | 2,148,419 | 0.37% |
|  | En-Spm | - | - | 0.00% | - | - | 0.00% |
|  | MULE-MuDR | 4,628 | 3,265,000 | 0.96% | 5,211 | 2,843,971 | 0.49% |
|  | PiggyBac | - | - | 0.00% | - | - | 0.00% |
|  | Tourist/Harbinger | 1,313 | 584,355 | 0.17% | 1,834 | 436,523 | 0.08% |
|  | Other (Mirage, P-element, Transib) | - | - | 0.00% | - | - | 0.00% |
| **Rolling-circles** |  | 7,846 | 3,923,495 | 1.15% | 5,684 | 2,468,239 | 0.43% |
| **Unclassified** |  | 299,555 | 112,210,144 | 32.98% | 616,342 | 174,026,050 | 29.98% |
| **Total interspersed repeats** |  |  | 163,440,001 | 48.03% |  | 350,922,063 | 60.46% |
| **Small RNA** |  | 624 | 3,258,780 | 0.96% | 16,795 | 5,040,471 | 0.87% |
| **Satellites** |  | - | - | 0.00% | - | - | 0.00% |
| **Simple repeats** |  | 79,978 | 2,981,502 | 0.88% | 170,520 | 7,089,649 | 1.22% |
| **Low complexity** |  | 16,168 | 764,505 | 0.22% | 25,007 | 1,234,954 | 0.21% |

**Supplementary Table 3**. Whole-genome duplication and speciation peaks analysis by Ks value

| **Species 1** | **Species 2** | **WGD peak** | **speciation peak** |
| --- | --- | --- | --- |
| ***Bauhinia*** | ***Bauhinia*** | 0.25 |  |
|  | *Cercis* |  | 0.20 |
|  | *Medicago* |  | 0.90 |
|  | *Phaseolus* |  | 0.80 |
|  | *Quillaja* |  | 0.75 |
|  | *Senna* |  | 0.60 |
|  | *Sindora* |  | 0.65 |
| ***Cercis*** | ***Cercis*** | 2.00 |  |
|  | *Medicago* |  | 0.80 |
|  | *Phaseolus* |  | 0.70 |
|  | *Quillaja* |  | 0.65 |
|  | *Senna* |  | 0.50 |
|  | *Sindora* |  | 0.55 |
| ***Medicago*** | ***Medicago*** | 1.00 |  |
|  | *Phaseolus* | 0.90 | 0.75 |
|  | *Quillaja* |  | 1.10 |
|  | *Senna* |  | 0.90 |
|  | *Sindora* |  | 0.95 |
| ***Phaseolus*** | ***Phaseolus*** | 0.80 |  |
|  | *Quillaja* |  | 1.00 |
|  | *Senna* |  | 0.80 |
|  | *Sindora* |  | 0.90 |
| ***Quillaja*** | ***Quillaja*** | 0.30 |  |
|  | *Senna* |  | 0.80 |
|  | *Sindora* |  | 0.85 |
| ***Senna*** | ***Senna*** | 0.65 |  |
|  | *Sindora* |  | 0.70 |
| ***Sindora*** | ***Sindora*** | 0.60 |  |
